# A high-density genome-wide association with absolute blood monocyte count in domestic sheep identifies novel loci

**DOI:** 10.1371/journal.pone.0266748

**Published:** 2022-05-06

**Authors:** Ryan D. Oliveira, Michelle R. Mousel, Michael V. Gonzalez, Codie J. Durfee, Kimberly M. Davenport, Brenda M. Murdoch, J. Bret Taylor, Holly L. Neibergs, Stephen N. White

**Affiliations:** 1 Department of Veterinary Microbiology & Pathology, Washington State University, Pullman, Washington, United States of America; 2 USDA-ARS Animal Disease Research, Pullman, Washington, United States of America; 3 Allen School for Global Animal Health, Washington State University, Pullman, Washington, United States of America; 4 Center for Applied Genomics, Children’s Hospital of Philadelphia, Philadelphia, PA, United States of America; 5 Department of Animal, Veterinary, and Food Science, University of Idaho, Moscow, ID, United States of America; 6 Center for Reproductive Biology, Washington State University, Pullman, WA, United States of America; 7 USDA-ARS Range Sheep Production Efficiency Research, Dubois, Idaho, United States of America; 8 Department of Animal Sciences, Washington State University, Pullman, WA, United States of America; University of Delaware, UNITED STATES

## Abstract

Monocytes are a core component of the immune system that arise from bone marrow and differentiate into cells responsible for phagocytosis and antigen presentation. Their derivatives are often responsible for the initiation of the adaptive immune response. Monocytes and macrophages are central in both controlling and propagating infectious diseases such as infection by *Coxiella burnetii* and small ruminant lentivirus in sheep. Genotypes from 513 Rambouillet, Polypay, and Columbia sheep (*Ovis aries*) were generated using the Ovine SNP50 BeadChip. Of these sheep, 222 animals were subsequently genotyped with the Ovine Infinium^®^ HD SNP BeadChip to increase SNP coverage. Data from the 222 HD genotyped sheep were combined with the data from an additional 258 unique sheep to form a 480-sheep reference panel; this panel was used to impute the low-density genotypes to the HD genotyping density. Then, a genome-wide association analysis was conducted to identify loci associated with absolute monocyte counts from blood. The analysis used a single-locus mixed linear model implementing EMMAX with age and ten principal components as fixed effects. Two genome-wide significant peaks (p < 5x10^-7^) were identified on chromosomes 9 and 1, and ten genome-wide suggestive peaks (p < 1x10^-5^) were identified on chromosomes 1, 2, 3, 4, 9, 10, 15, and 16. The identified loci were within or near genes including *KCNK9*, involved into cytokine production, *LY6D*, a member of a superfamily of genes, some of which subset monocyte lineages, and *HMGN1*, which encodes a chromatin regulator associated with myeloid cell differentiation. Further investigation of these loci is being conducted to understand their contributions to monocyte counts. Investigating the genetic basis of monocyte lineages and numbers may in turn provide information about pathogens of veterinary importance and elucidate fundamental immunology.

## Introduction

Monocytes are bone marrow-derived immune cells that circulate in the blood with a short lifespan, typically days [1]. They have traditionally been grouped in the mononuclear phagocyte system that includes macrophages and dendritic cells, though this grouping is based on historical studies of function and likely bridges several independent lineages [2]. Monocytes are an essential component in the immune response to a variety of pathogens [3], and they can be a source of some tissue resident macrophages and dendritic cells [4]. They also support differentiation of various T cell lineages in lymph nodes [5, 6], emphasizing their importance in both innate and adaptive immunity.

Monocytes can differentiate along multiple lineages to produce several categories of cells which are still being characterized [7, 8]. In brief, studies in mice imply that monocytes initially derive from a common myeloid progenitor, which becomes either a granulocyte-monocyte progenitor or monocyte-dendritic cell progenitor, and each lineage gives rise to separate monocyte subsets as distinguished with RNA profiles [7, 9]. Studies in human-derived cells suggest a similar multilineage differentiation of monocytes [10, 11].

After leaving the marrow, monocytes enter the circulation and can migrate to lymphoid and non-lymphoid tissues. Canonically, they are characterized as classical, nonclassical, and intermediate subsets in humans based on CD14 and CD16 expression profiles [12], while in mice they are similarly divided based on Ly6C expression [13]. Generally, classical monocytes enter tissues to aid in inflammation, while nonclassical monocytes are thought to patrol the vascular surfaces [14, 15]. Classical monocytes may terminally differentiate into macrophages or dendritic cells [16].

The concentration of monocytes in circulation, under normal conditions, stays within a consistent reference interval that is often measured in complete blood counts in the clinical setting [17]. In most studied species, monocyte elevations occur under conditions of inflammation. In humans, chronic elevations may be a biomarker of cardiovascular disease [18]. In otherwise healthy middle-aged and elderly adults, higher levels of circulating monocytes are associated with increased risk of cancer and mortality [19].

In sheep, monocytes are important in both the propagation and control of several infectious diseases. For example, small ruminant lentivirus, which causes a multisystemic inflammatory disease in sheep and goats [20], has been known for decades to use the monocyte system to disseminate through the bloodstream [21]. Viral replication in monocytes is tied to their maturation into phagocytes [22], and dendritic cells are an important site of infection as well [23, 24]. Similarly, *Coxiella burnetii*, an ubiquitous bacterial infection of sheep and goats that also causes the human disease Q fever [25], subverts classical phagocytosis to infect and replicate in the mononuclear phagocyte system [26]. *C*. *burnetii* actively replicates in monocytes and their derived macrophages [27, 28], including cell lines created from circulating monocytes [29].

Identifying the genetic influences underlying the amount of available circulating monocytes is paramount to characterizing the immune response to diseases influenced by their presence. Genome-wide association studies (GWAS) are a useful methodology employed to identify sites in the genome associated with phenotypes such as monocyte counts. Genotyping a wide array of natural variations across the genome such as single nucleotide polymorphisms (SNPs) or insertion-deletions (indels) followed by a GWAS can identify loci associated with a trait of interest. The genetic influences on monocyte count in humans have been explored extensively using GWAS and related methods. Variations in several leukocyte receptors and other proteins associated with leukocytic differentiation and function have been observed to partially account for circulating monocyte count and occasionally other circulating leukocytes in humans [30–36]. From these studies, variations in or near *ITGA4* and *LPAR1* have been found to be important in multiple human populations [33–35].

The effect of genetic variation on monocyte count in domestic animals has not been studied despite the important influence of the mononuclear phagocyte system in disease. Additionally, the ability of a GWAS to identify loci associated with a trait can be assisted by imputation, a process by which an algorithm estimates genotypes from a lower density genotyping profile to a higher density profile through principles of chromosomal linkage. This is performed when existing high-density (HD) genotypes from a reference population are available to accurately estimate the missing HD genotypes in a new population. This study uses imputed HD genotypes in a GWAS to identify loci and putative candidate genes that are associated with circulating monocyte count in sheep.

## Materials and methods

### Populations and phenotypes

#### Absolute monocyte count analysis

Whole blood was obtained by jugular venipuncture into EDTA-coated vacutainer tubes from ewes (*Ovis aries*) at the U.S. Sheep Experiment Station in Dubois, Idaho as part of a previous study [37], using methods previously described [38]. For the present study, a subset of 513 sheep was selected from ewes of Columbia (N = 67), Polypay (N = 196), and Rambouillet (N = 250) breeds and of ages between 1 and 5 years. These three breeds were chosen based on their divergence of growth, reproductive, immune, and blood traits, and previous genetic studies [37, 39–44], and because they are among the most common production breeds in the United States. The Columbia and Polypay breeds were derived from the Rambouillet breed [45, 46], and close genetic relationships still exist between these breeds [46]. Complete blood counts were performed on this subset as previously described [37]. Briefly, absolute monocyte counts were obtained as part of complete blood counts performed by Phoenix Labs, Inc. (Everett, Washington, USA) within approximately 24 hours of the time of collection, with reference values provided by Phoenix Labs, Inc.

#### Ethics statement

All animal care and handling procedures were reviewed and approved by the Washington State University Institutional Animal Care and Use Committee (Permit Numbers: 3171, 4885, and 4594) and/or by the U.S. Sheep Experiment Station Animal Care and Use Committee (Protocol Numbers: 04–14, 10–07, 15–04, 15–05). All efforts were made to minimize any discomfort during blood collection.

### Genotyping methods

#### Genotyping with Ovine SNP50 BeadChip

The process of genotyping and imputation is summarized in S1 Fig. Genotyping at a lower density was performed for the study population of 513 animals as described in a previous study [37]. Briefly, DNA was isolated using the Invitrogen GeneCatcher^TM^ gDNA 3–10 ml Blood Kit using the manufacturers’ instructions (Life Technologies, Carlsbad, CA). Genotyping services were provided by Geneseek Inc. (Lincoln, NE) using the OvineSNP50 BeadChip (Illumina Inc., San Diego, CA) with a set of 54,977 SNPs designed by the International Sheep Genome Consortium [47].

#### Genotyping with Ovine Infinium^®^ HD SNP BeadChip

Using the same DNA, a subset of 222 sheep from the lower density dataset (Columbia N = 33, Polypay N = 80, Rambouillet N = 109) were genotyped again using the Ovine Infinium^®^ HD SNP BeadChip, which uses a set of 606,006 SNPs also designed by the International Sheep Genome Consortium [48]. A separate group of 258 sheep (Columbia N = 33, Polypay N = 140, Rambouillet N = 85) also from the U.S. Sheep Experiment Station, had blood drawn [49] with DNA extraction as previously described [38] but without complete blood counts. These sheep were also genotyped with the HD SNP BeadChip to aid in a reference panel for genotype imputation.

#### Genotype imputation

Unphased genotype data were converted from PLINK v1.9.ped format [50, 51] to variant call format with a script in R v4.0.2 using the data.table v1.13.2 and R.utils v2.10.1 packages [52]. As part of pre-imputation quality control, loci without an assigned chromosome or base pair position, loci with a call rate lower than 95% from either the lower density (2,407 SNPs) or HD array (25,183 SNPs), and individuals with a call rate lower than 95% from either array (0 individuals) were removed from the dataset. Genotypes of the sheep were designated into two major sets based on genotyping density: a to-be-imputed group of 291 sheep genotyped by the Ovine SNP50 BeadChip (Columbia N = 34, Polypay N = 116, Rambouillet N = 141), and a reference panel of 480 sheep genotyped by the Ovine Infinium^®^ HD SNP BeadChip (Columbia N = 66, Polypay N = 220, Rambouillet N = 194) created by merging the groups of 222 and 258 sheep previously described.

Each set was in turn separated into its three component breeds, and imputation of each low-density breed group was performed using its corresponding breed group in the reference panel. Imputation occurred in two major steps. First, the reference panel for each breed was phased with imputation of SNP50-exclusive loci using Beagle v5.1 [53, 54]. Once all genotypes had been determined in these groups, they were used as reference panels for breed-specific imputation and phasing of the low-density genotypes, again using Beagle v5.1 [53, 54]. Imputation accuracies were predicted by five-fold cross-validation in the reference population and were 0.705 for Columbia sheep, 0.735 for Polypay sheep, and 0.680 for Rambouillet sheep.

### Statistical analysis

#### Genome-wide association analysis with absolute monocyte count

Genome-wide association with the absolute monocyte count was performed using SNP & Variation Suite (SVS) version 8.9.0 (Golden Helix, Inc., Bozeman, MT, US). Variants with minor allele frequency below 1% were removed (45,263 SNPs). A Hardy-Weinberg test of equilibrium test was performed, and 768 SNPs at extreme skew (p < 1 x 10^−80^) were removed. After all quality control measures, 542,255 SNPs remained for absolute monocyte count analysis. Initially chosen fixed effects included age and breed. Thirty principal components were initially generated from the final curated set of genotypes using SVS. The ten principal components with the highest eigenvalues accounted for 84.4% of the variation explained by all thirty, and as such the other principal components were dropped from the analysis (S1 Table). As the principal components were found to account for breed (S2–S4 Figs), breed was removed as a fixed effect. The association model used was a single-locus mixed linear model implementing EMMAX [55] using age and varying amounts of principal components as fixed effects with a calculated identity-by-state matrix as a random effect. A threshold of p < 5x10^-7^ was used for genome-wide significance, and a threshold of p < 1x10^-5^ was used for genome-wide suggestive evidence [56]. Genomic inflation factor (pseudo lambda) was calculated using a custom script for SVS hosted on the Golden Helix website [57]. The Manhattan plot was generated using a modification of a script developed by Pagé Goddard in R (https://github.com/pcgoddard/Burchardlab_Tutorials/wiki/GGplot2-Manhattan-Plot-Function, viewed on December 27, 2018). The Quantile-Quantile (Q-Q) plots were constructed using the qqman v0.1.8 package in R [52, 58].

## Results

Absolute monocyte counts measured in the 513 sheep varied from 0 to 880 per microliter, with a mean of 205.7, a median of 190, and a standard deviation of 125.0 (S2 Table). More detailed distribution characteristics are listed in S3 Table. The monocyte count reference interval typically ranges up to 750 per microliter in sheep [59, 60], and only one animal exceeded this reference interval at 880 per microliter. No study sheep were noted to be clinically ill during sampling. The calculated genomic inflation factor (pseudo lambda) for the final genome-wide association analysis was 1.01.

**Fig 1:**
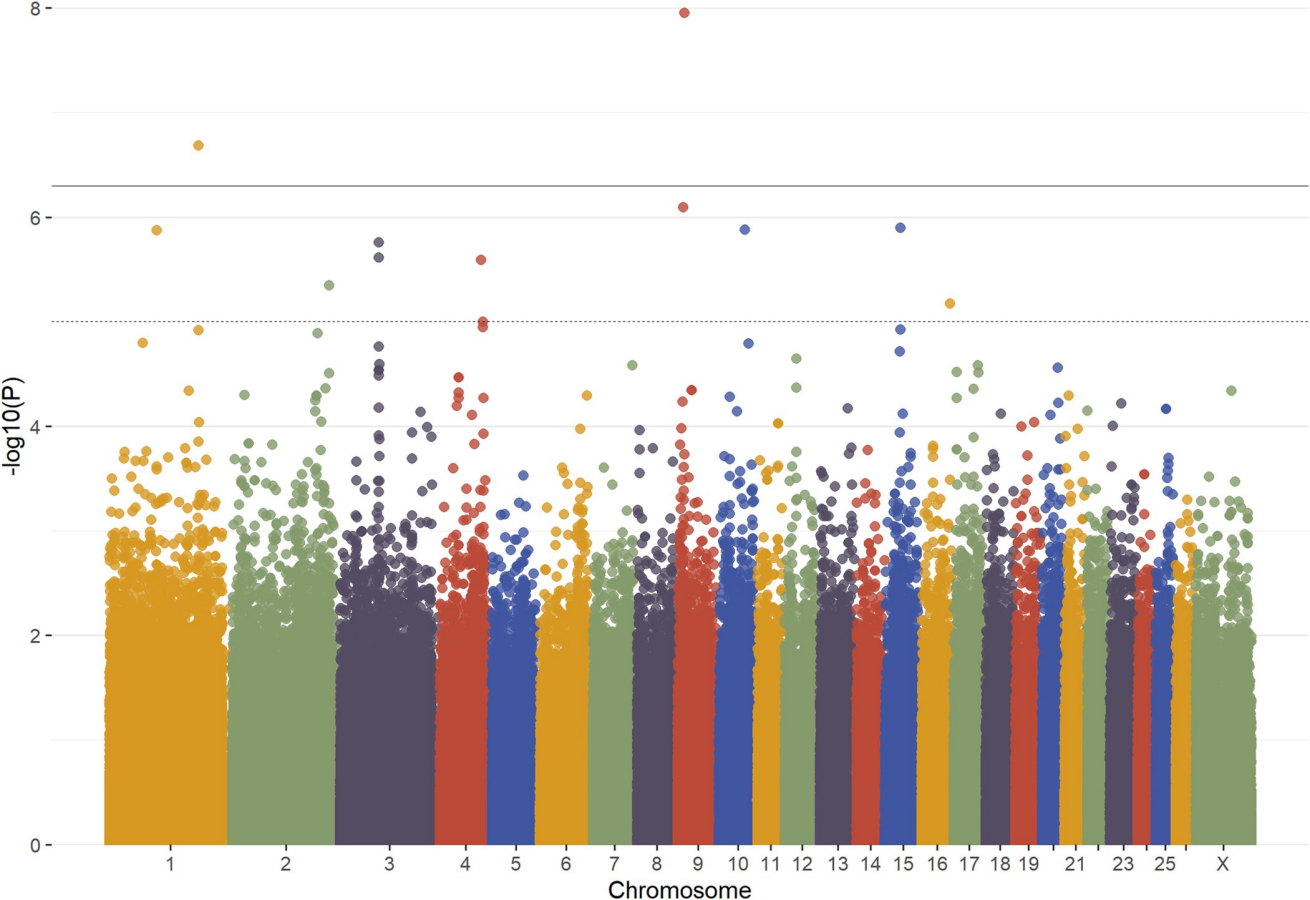
Manhattan plot of absolute monocyte count GWAS. The Manhattan plot displays all nominal p values from the GWAS for absolute monocyte count by chromosomal position. Representative data from the additive genetic analysis is shown. The dotted and solid lines respectively denote p values of 1x10^-5^ (genome-wide suggestive) and 5x10^-7^ (genome-wide significant).

As can be seen in the Manhattan plot (Fig 1), two loci had p values below the genome-wide significance threshold, while ten loci passed the genome-wide suggestive threshold. Detailed information on genome-wide significant SNPs is in Table 1, and more information on genome-wide suggestive SNPs is in Table 2. A Q-Q plot for this analysis is given in S5 Fig. A second single-locus analysis was performed with the same terms as the first but with the five SNPs with the lowest p values as fixed effects (S6 Fig). The allele frequencies by breed for each the genome-wide significant and suggestive SNP are in S4 Table.

**Table 1:** Genome-wide significant single nucleotide polymorphisms (SNPs) associated with absolute monocyte count.

Chr	refSNP	Variant type	Position bp	A1	A2	MAF	P-value	Genes within 100 Kb
9	*rs401041089*	intergenic	18,019,166	G	A^+^	0.011	1.10x10^-8^	Potassium two pore domain channel subfamily K member 9 (*KCNK9*)^2^
1	*rs428401450*	intergenic	238,399,139	G	T^+^	0.151	2.05x10^-7^	No genes within 100 Kb

All positions use the *Oar rambouillet v1*.*0* reference genome unless otherwise indicated.

^+^Allele associated with higher absolute monocyte count.

^1^Located upstream from gene.

^2^Located downstream from gene.

**Table 2:** Genome-wide suggestive single nucleotide polymorphisms (SNPs) associated with absolute monocyte count.

Chr	refSNP	Variant type	Position bp	A1	A2	MAF	P-value	Genes within 100 Kb
9	*rs425174370*	intergenic	15,830,787	T	G^+^	0.014	7.98x10^-7^	Lymphocyte antigen 6 family member D (*LY6D*)^1^, Ly6/neurotoxin-like protein 1 (*LYNX1*)^1^, Ly6/PLAUR domain containing 2 (*LYPD2*)^1^, Secreted Ly6/LAUR domain containing 1(*SLURP1*)^1^, Cytochrome P450 family 11 subfamily B polypeptide 1 (*CYPB11B1*)^1^, Thioesterase superfamily member 6 (*THEM6*)^1^, Prostate stem cell antigen (*PSCA*)^1^, *ENSOARG00020013707*^2^, Glycosylphosphatidylinositol anchored molecule like (*GML*) ^2^
15	*rs399452398*	intergenic	41,848,001	C	T^+^	0.038	1.26x10^-6^	Spondin 1 (*SPON1*)^2^
10	*rs429734375*	intergenic	69,691,108	A	G^+^	0.260	1.30x10^-6^	No genes within 100 Kb
1	*rs421879522*	intergenic	125,531,997	C	T^+^	0.065	1.33x10^-6^	Family with sequence similarity 78 member B (*FAM78B*)^1^, Uridine-cytidine kinase 2 (*UCK2*)^2^, Aldehyde dehydrogenase 9 family member A1 (*ALDH9A1*)^2^
3	*rs428909416*	intron	100,745,902 or 96,180,662	G^+^	A	0.415	1.74x10^-6^	High mobility group nucleosome binding domain 1 (*HMGN1*)^1^, Exocyst complex component 6B (*EXOC6B*)
3	*rs414400434*	intron	100,784,103	T^+^	C	0.485	2.42x10^-6^	Exocyst complex component 6B (*EXOC6B*)
4	*rs399619443*	intergenic	100,335,490, 109,357,840, or 109,444,337	C^+^	T	0.075	2.55x10^-6^	Cholinergic receptor muscarinic 2 (*CHRM2*)^1^
2	*rs418310516*	intergenic	253,537,862	C	A^+^	0.074	4.49x10^-6^	No genes within 100 Kb
16	*rs427185509*	intergenic	75,515,758	T	C^+^	0.025	6.65x10^-6^	No genes within 100 Kb
4	*rs423783355* ^*a*^	intergenic	104,754,549	A	G^+^	0.033	9.97x10^-6^	Olfactory receptor family 6 subfamily V member 1 (*OR6V1*)^1^^,^ Kell metallo-endopeptidase (*KEL*)^2^, Transient receptor potential cation channel subfamily V member 5 (*TRPV5*)^2^, LLLL and CFNLAS motif containing 1 (*LLCFC1*)^2^

All positions use the *Oar rambouillet v1*.*0* reference genome unless otherwise indicated. Loci with multiple positions listed mapped to multiple positions in Ensembl Release 104.

^+^Allele associated with higher absolute monocyte count.

^1^Located upstream from gene.

^2^Located downstream from gene. ^a^The SNP *rs423783355* did not map to the *Oar rambouillet v1*.*0* genome, so information from the *Oar v3*.*1* genome is used for this SNP.

## Discussion

The advent of high-density GWAS has provided the potential to improve scientific understanding of the genetic influences behind many important health and developmental traits [61], including those in the livestock industry. Previous GWAS in sheep have identified genetic variants underlying factors important to agriculture such as litter size [62], growth and meat production [63], and wool production [64]. Similar analyses have also shed light on the genetic architecture underpinning disease susceptibility in sheep [38, 42, 65] and can highlight genes not previously known to be involved with the assessed pathogen in a species [38]. Analyzing circulating levels of monocytes, an important immune effector cell in sheep diseases [21–23, 29], has a similar potential to benefit animal agriculture and expand on knowledge of genes associated with immune functions. Thus, we performed the first GWAS for circulating monocytes in sheep, associating genotypes imputed to high density in sheep with absolute monocyte counts. The breed imputation accuracies are mildly lower than initially reported accuracies in sheep [66], which may reflect the relatively limited availability of reference animals in this study. As a result, the findings must be interpreted in light of this limitation, which may affect their reliability. GWAS findings should be validated in an independent population to prevent overfitting and rule out spurious findings, and this is especially important as this study was limited somewhat by sample size and correspondingly low imputation accuracy. To the authors’ knowledge, however, this is the first association study utilizing imputation in Columbia, Polypay, and Rambouillet sheep. The model from our analysis had a genomic inflation factor of 1.01, indicating minimal test statistic inflation, and identified two SNPs of genome-wide significance and ten genome-wide suggestive SNPs across eight autosomes as listed in Tables 1 and 2. In this discussion, we delve into the potential importance of some of the associated loci, particularly with regard to nearby genes and how those genes may impact circulating monocyte count, though a link was not apparent with every locus.

Two genome-wide significant SNPs were identified. One SNP (*rs401041089*; p = 1.10 x 10^−8^; Table 1) is approximately 60 kilobases downstream of *KCNK9*, a gene encoding a pH-dependent potassium channel. This channel, also called TASK3, is constitutively expressed by T lymphocytes and affects downstream functions of T cell-receptor activation *in vitro*, including secretion of the proinflammatory cytokines IFNγ and IL2 [67]. IFNγ is linked in particular to the monocyte-macrophage lineage, with these cells both producing and becoming activated in response to IFNγ [68]. Monocyte precursors in murine bone marrow express the IFNγ receptor IFNγR1 [69]. IFNγ is thought to shift hematopoiesis in favor of monocytes during inflammation based on models of infectious disease in mice [70, 71]. TASK3 is also linked with apoptosis in cultured neurons [72] and human gastric adenocarcinoma cell lines [73]. The other significant SNP (*rs428401450*; p = 2.05 x 10^−7^) was not within 100 kilobases of any recognized genes in the *Oar rambouillet v1*.*0* assembly [74–76], though its association is suggested by the presence of three SNPs within 50 kilobases with p values less than 1 x 10^−3^. However, *rs428401450* is approximately 114 kilobases upstream of the gene *GOLIM4*, which encodes a type II Golgi-resident protein considered to play a role in cell proliferation and apoptosis in some neoplastic cell lines [77, 78]. Additionally, the genomic locus where the SNP is found has a chromatin state consistent with an active promoter in tissue from the spleen [79], and the SNP is within 100 bases of chromatin consistent with an active promoter in alveolar macrophages [80]. While a gene is not identified in any closer vicinity than that of *GOLIM4*, it is not uncommon for GWAS findings to reside within “gene deserts”, with one studying estimating that nearly half of published SNPs associated with disease discovered by GWAS are not within or near genes [81]. Such areas may still contain regulatory elements that act on distant genes utilizing the three-dimensional structure of chromatin [81, 82], and projects that annotate genomes and elucidate chromatin structure are expected to reveal more information about these regions. For example, annotation in sheep is an ongoing effort with recent developments by the Functional Annotation of Animal Genomes (FAANG) consortium [79]. The FAANG project aims to map functional elements in the genomes of several domesticated animal species [83], and the ovine FAANG project has published multiple papers describing functional elements in the recent *Oar rambouillet v1*.*0* genome [75, 79, 80].

Regarding the genome-wide suggestive SNPs, one locus (*rs425174370*; p = 7.98 x 10^−7^; Table 2) resides within a gene cluster on OAR9 including *GML*, *LY6D*, *LYNX1*, *LYPD2*, and *SLURP1*, which are members of the lymphocyte antigen-6/urokinase-type plasminogen activator receptor (Ly6/uPAR) family of genes [84, 85]. Ly6/uPAR genes are associated with stem cells and are particularly implicated in immune cell differentiation and clearance of cancer [84], and expression of the family member *Ly6C* defines classical and non-classical monocyte subsets in mice [13, 14, 86]. The gene family is conserved across species, though some gene family members are species-specific [85], and human monocyte subsets are classified as classical, non-classical, or intermediate by a different system [12]. While such a classification scheme does not exist in sheep, the association of loci within this gene cluster with absolute monocyte count could indicate such a system exists in sheep and/or that members of the LY6/uPAR family play a role in monocyte differentiation; establishing such subsets could be an exciting development in sheep monocyte physiology that deserves to be explored further.

Two genome-wide suggestive loci are on OAR3 (*rs428909416*; p = 1.74 x 10^−6^ and *rs414400434*; p = 2.42x10^-6^; Table 2), with *rs428909416* mapping to two positions in *Oar rambouillet v1*.*0* in Ensembl release 104 [74, 75, 87]. If the position at 100,745,902 is used for *rs428909416*, both SNPs are within *EXOC6B*, which encodes part of a multimeric protein called the exocyst, which is highly expressed in mast cells [88] and has a general role in exocytosis [89]. However, using the alternate position for *rs428909416* places the locus close to the ENSOART00020002401.1 transcript on Ensembl [87], and a pairwise comparison of its 360 bp cDNA sequence had 100% nucleotide sequence identity with a predicted transcript from *HMGN1* (XM_027963980.1) using a BLASTn search [90]. *HMGN1* codes for a chromatin accessibility regulator closely linked with myeloid differentiation, being highly expressed in hematopoietic stem cells and progenitor cells but losing expression in differentiated myeloid cells [91]. In addition, extracellular product acts as an alarmin that favors a Th1 type response [92], an inflammatory pattern associated with IFNγ [93] and a strong cell-mediated response including macrophages [94].

Finally, a genome-wide suggestive SNP on OAR4 (*rs423783355*; p = 9.97 x 10^−6^; Table 2) does not map to *Oar rambouillet v1*.*0* but maps close to several annotated genes in *Oar v3*.*1* in NCBI [95] and near a long non-coding RNA in Ensembl [87]. Of particular interest, it is approximately 15 kilobases downstream of *KEL*, which encodes the Kell blood antigen group [96]. While best characterized as a glycoprotein determining blood groups in erythrocytes, Kell is also expressed on myeloid progenitor cells and monocytes [97, 98].

While no previous GWAS with monocyte counts have been performed in sheep, similar studies have been done for humans, with the genes *ITGA4* and *LPAR1* implicated in multiple study populations [33–35]. These genes were not associated with absolute monocyte count in this study. While this study identifies several loci that have not been previously associated with the number of circulating monocytes in humans, one associated locus is near several genes in the Ly6/uPAR family, members of which are associated with immune cell differentiation and monocyte subclassification in mice [13, 85]. These findings shed new light on monocyte differentiation in sheep and can potentially help characterize the immune response associated with important infectious diseases of this species. These findings will be further investigated to determine their associated causal variant(s) and assess their validity.

## Conclusions

This study is the first to perform a genome-wide association with a measure of circulating monocytes in sheep. Several significant and suggestive loci, all novel loci compared with those identified in human studies, were identified and are located near genes associated with monocyte subset differentiation, cytokine production, cell-mediated immunity, and hematopoiesis. Characterizing the genetic influences underlying monocyte counts in sheep may also have value in investigating the susceptibility to diseases that closely involve the monocyte-macrophage system such as *Coxiella burnetii* and ovine lentivirus. More research such as fine mapping is needed to identify what causal variants may be in linkage disequilibrium with the identified loci, and these functional mutations should be validated in an independent population. Fully characterizing these loci has the potential to benefit veterinary medicine, producers, and expand knowledge of immunologic development.

## Supporting information

S1 Fig Flowchart for genotype imputation. A summary of how genotype measurement and imputation were conducted.
(TIF)

S2 Fig Principal components 1 and 2 by breed. The first and second principal components are plotted against each other for the 513 study sheep, with each point color-coded to show breed.
(TIFF)

S3 Fig Principal components 2 and 3 by breed. The second and third principal components are plotted against each other for the 513 study sheep, with each point color-coded to show breed.
(TIFF)

S4 Fig Principal components 1 and 3 by breed. The first and third principal components are plotted against each other for the 513 study sheep, with each point color-coded to show breed.
(TIFF)

S5 Fig Quantile-quantile plot for the main mixed model analysis. The quantile-quantile (QQ) plot for the mixed model used in the main analysis in the paper.
(TIF)

S6 Fig Quantile-quantile plot for an adjunct mixed model analysis. The quantile-quantile (QQ) plot for an additional mixed model analysis run after the primary one. The five SNPs with lowest p values were added to the first model as fixed effects.
(TIF)

S1 Table Principal components by study ID. The ten principal components used in the analysis matched to sheep study ID.
(DOCX)

S2 Table Monocyte counts by study ID. The absolute monocyte count matched to sheep study ID.
(DOCX)

S3 Table Distribution characteristics of monocyte counts. The distribution characteristics for the absolute monocyte counts are summarized.
(DOCX)

S4 Table Allele frequencies of the top SNPs by breed. ^+^Allele associated with higher absolute monocyte count.
(DOCX)
