# Supplementary figures and images for: A high-density genome-wide association with absolute blood monocyte count in domestic sheep identifies novel loci
Source: PLoS One. 2022 May 6;17(5):e0266748. doi: 10.1371/journal.pone.0266748 (PMC9075649; doi:10.1371/journal.pone.0266748)

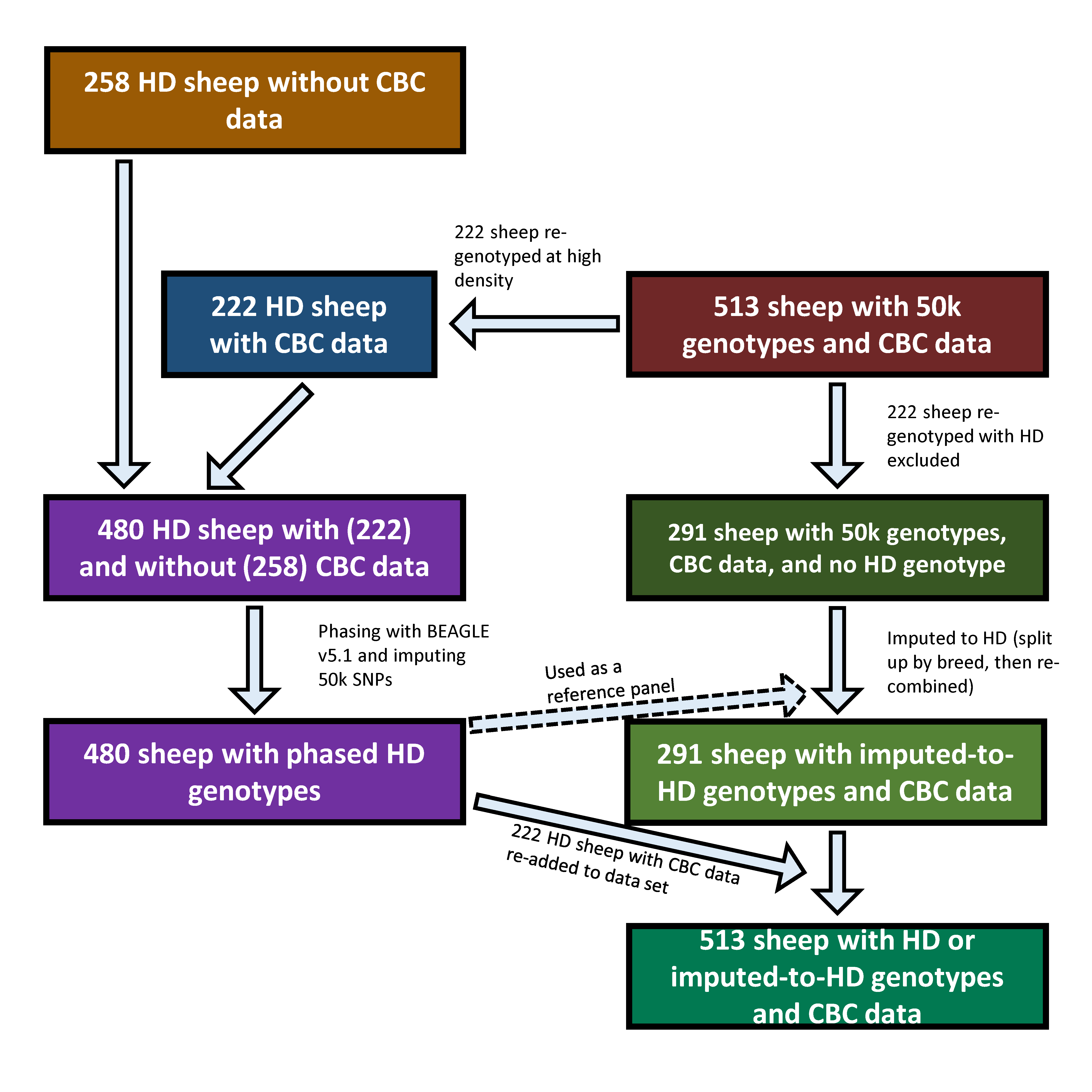

Supplement: S1 Fig — A summary of how genotype measurement and imputation were conducted. (TIF) [file pone.0266748.s001.tif]

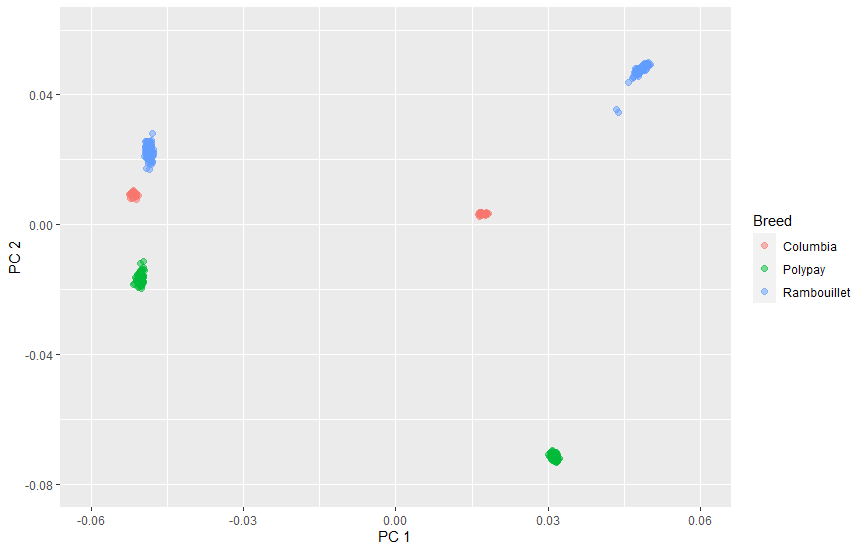

Supplement: S2 Fig — The first and second principal components are plotted against each other for the 513 study sheep, with each point color-coded to show breed. (TIFF) [file pone.0266748.s002.tiff]

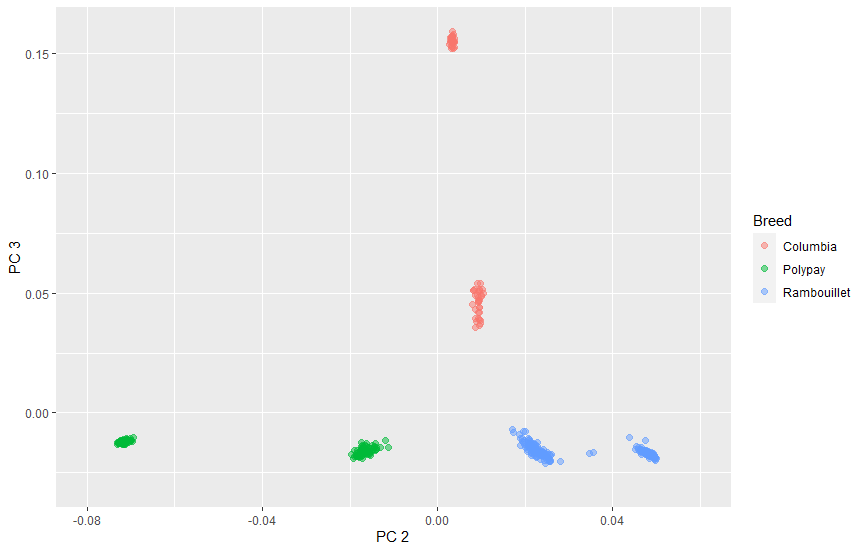

Supplement: S3 Fig — The second and third principal components are plotted against each other for the 513 study sheep, with each point color-coded to show breed. (TIFF) [file pone.0266748.s003.tiff]

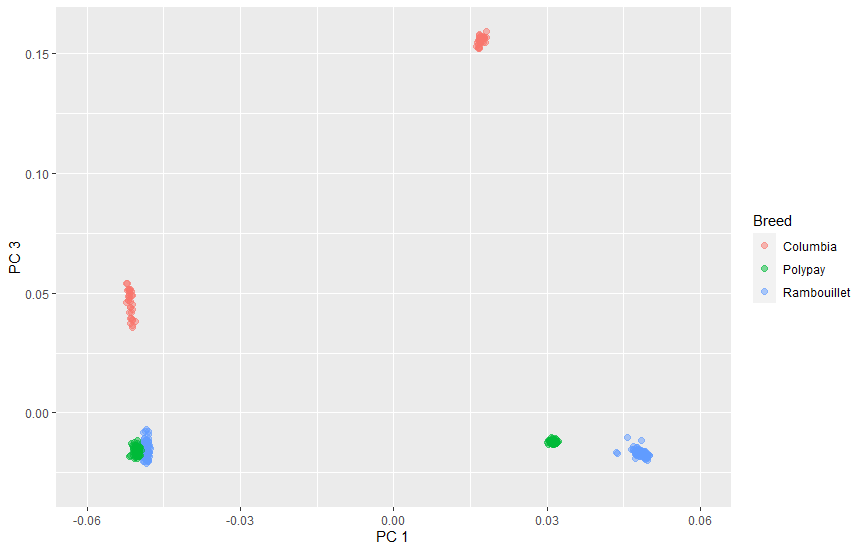

Supplement: S4 Fig — The first and third principal components are plotted against each other for the 513 study sheep, with each point color-coded to show breed. (TIFF) [file pone.0266748.s004.tiff]

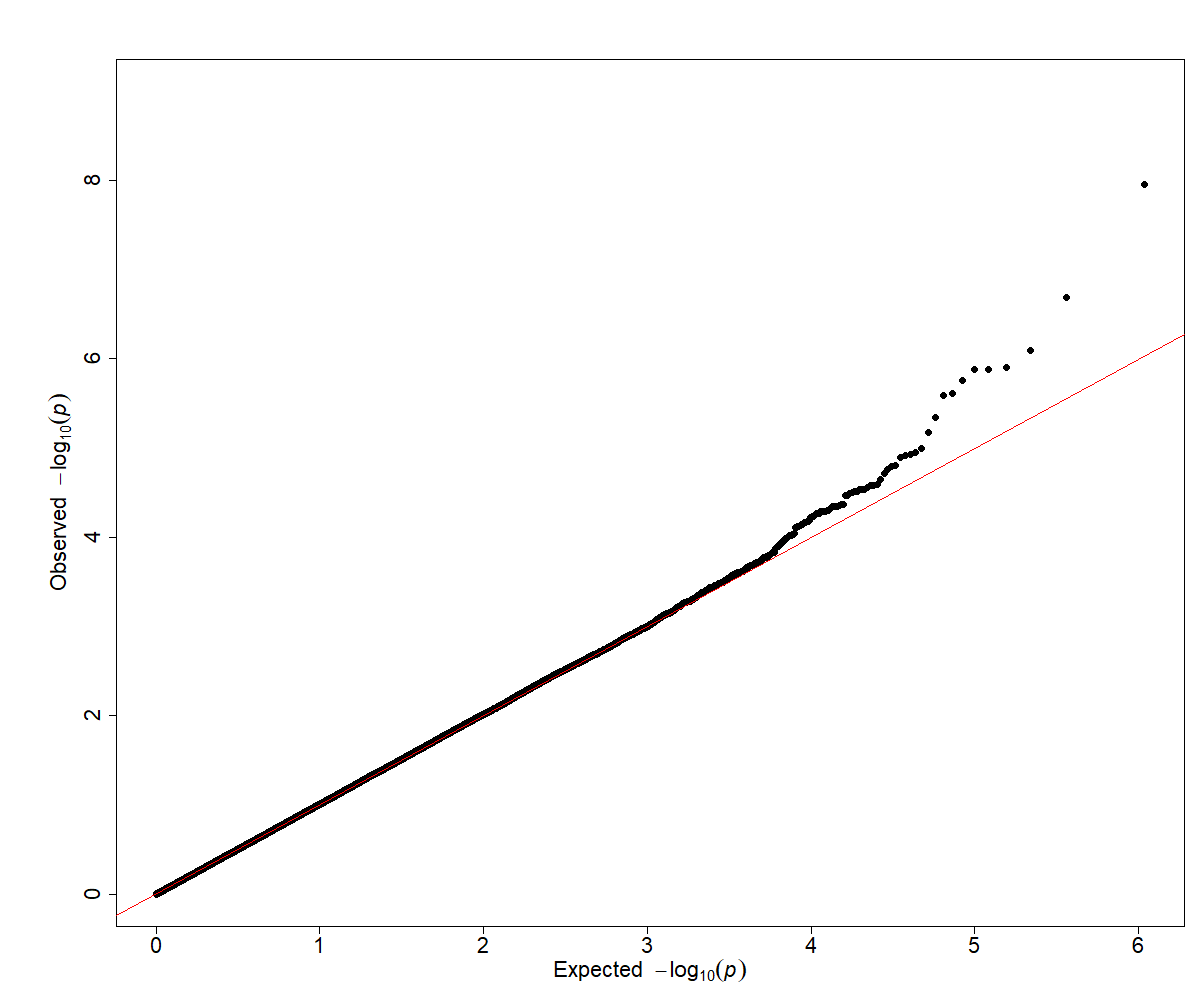

Supplement: S5 Fig — The quantile-quantile (QQ) plot for the mixed model used in the main analysis in the paper. (TIF) [file pone.0266748.s005.tif]

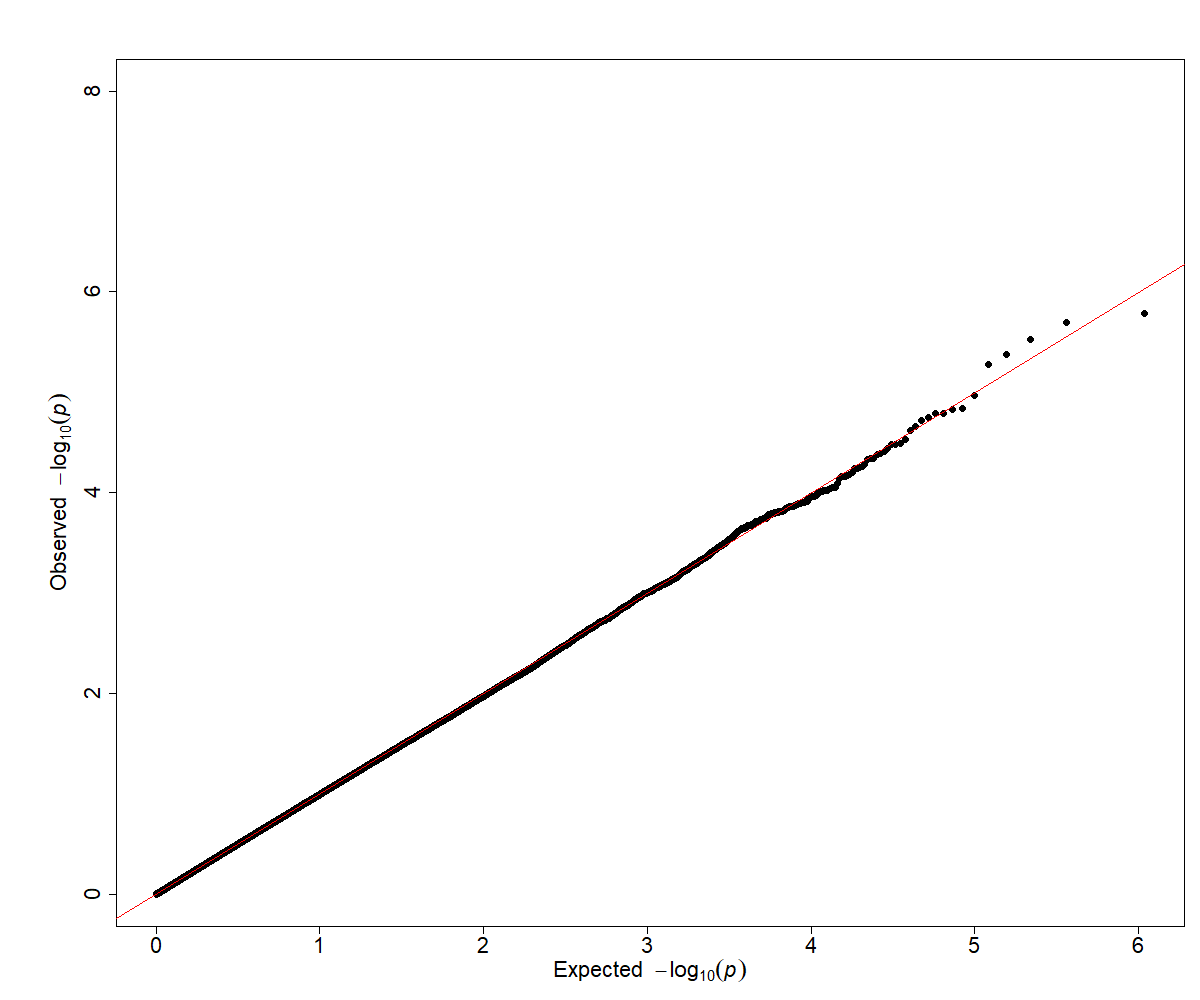

Supplement: S6 Fig — The quantile-quantile (QQ) plot for an additional mixed model analysis run after the primary one. The five SNPs with lowest p values were added to the first model as fixed effects. (TIF) [file pone.0266748.s006.tif]
