# Supplementary material for: A high-density genome-wide association with absolute blood monocyte count in domestic sheep identifies novel loci
Source: PLoS One. 2022 May 6;17(5):e0266748. doi: 10.1371/journal.pone.0266748 (PMC9075649; doi:10.1371/journal.pone.0266748)
Supplement: S1 Table — The ten principal components used in the analysis matched to sheep study ID. (DOCX) [file pone.0266748.s007.docx]

**Table S1. Principal components by study ID.**

| Sheep | PC 1 | PC 2 | PC 3 | PC 4 | PC 5 | PC 6 | PC 7 | PC 8 | PC 9 | PC 10 |
| --- | --- | --- | --- | --- | --- | --- | --- | --- | --- | --- |
| 1 | 0.016551 | 0.003716 | 0.152322 | -0.00412 | -0.04937 | -0.00085 | -0.0005 | -0.0008 | -0.00369 | 0.000729 |
| 2 | 0.016991 | 0.003354 | 0.154823 | -0.00369 | -0.05003 | -0.0019 | -0.00082 | -0.00052 | 0.002492 | -0.00205 |
| 3 | 0.017074 | 0.00319 | 0.155802 | -0.00301 | -0.05408 | -0.00265 | -0.00015 | -0.00224 | -0.0002 | -0.002 |
| 4 | 0.01718 | 0.003499 | 0.156505 | -0.00423 | -0.05517 | -0.00164 | 0.000485 | -0.00081 | -0.0003 | 0.000847 |
| 5 | 0.017644 | 0.003452 | 0.156546 | -0.00458 | -0.05649 | -0.0019 | 0.001578 | 0.000716 | -0.00126 | -0.00093 |
| 6 | 0.016812 | 0.002997 | \| 0.156818 \| \| --- \| | -0.00409 | -0.05589 | -0.00224 | 0.002001 | -0.00032 | 0.001198 | 0.001563 |
| 7 | 0.016782 | 0.003523 | 0.158271 | -0.00465 | -0.05683 | -0.00306 | 0.001404 | -0.00188 | 0.000981 | -0.00109 |
| 8 | 0.017825 | 0.003427 | 0.15703 | -0.00425 | -0.05559 | -0.00214 | 0.001308 | 0.000647 | -3.36E-05 | 0.001469 |
| 9 | 0.017496 | 0.003178 | 0.156767 | -0.00337 | -0.05591 | -0.00222 | -0.0023 | -0.00337 | 0.000152 | 0.001299 |
| 10 | 0.017404 | 0.003226 | 0.156465 | -0.00487 | -0.05314 | -0.00116 | -0.00238 | 0.000571 | -0.00206 | 0.00117 |
| 11 | 0.017026 | 0.003329 | 0.154246 | -0.00368 | -0.05352 | -0.00077 | 0.000525 | -0.00022 | 0.000849 | 3.88E-05 |
| 12 | 0.017659 | 0.003267 | 0.156823 | -0.00327 | -0.05726 | -0.00175 | 0.001004 | -0.0018 | 0.001696 | 0.000976 |
| 13 | 0.016716 | 0.00356 | 0.155056 | -0.00498 | -0.05353 | -0.00317 | -0.00066 | -0.00054 | -0.00142 | 0.001009 |
| 14 | 0.016861 | 0.003382 | 0.157754 | -0.00486 | -0.05644 | -0.00249 | 0.001051 | -2.55E-05 | -0.00057 | -0.00091 |
| 15 | 0.017846 | 0.003686 | 0.154614 | -0.0042 | -0.05213 | -0.00135 | -0.00036 | 0.000171 | -0.00028 | 0.000147 |
| 16 | 0.016718 | 0.003944 | 0.152891 | -0.00346 | -0.04949 | -0.00201 | 0.00029 | -0.00083 | -0.00206 | -0.00049 |
| 17 | 0.016449 | 0.003628 | 0.154588 | -0.00404 | -0.05267 | -0.00134 | 0.001313 | 0.000697 | 0.000235 | -0.00014 |
| 18 | 0.016553 | 0.002704 | 0.154137 | -0.00503 | -0.052 | -0.00129 | 0.000477 | 0.000739 | 0.000231 | 0.00081 |
| 19 | 0.016941 | 0.003417 | 0.157283 | -0.00394 | -0.05792 | -0.00119 | 0.000617 | 0.000227 | -7.60E-05 | 0.000752 |
| 20 | 0.01804 | 0.003268 | 0.156921 | -0.00315 | -0.05569 | -0.00044 | -0.00177 | -0.00048 | -0.00241 | 0.001773 |
| 21 | 0.016741 | 0.003193 | 0.152234 | -0.00456 | -0.04874 | -0.00183 | -0.00068 | -0.00104 | -0.0015 | -0.00124 |
| 22 | 0.016923 | 0.003497 | 0.15509 | -0.00344 | -0.05485 | -0.00177 | 0.000535 | -0.00112 | -0.00447 | -0.00148 |
| 23 | 0.016836 | 0.003759 | 0.155032 | -0.00442 | -0.05286 | -0.00177 | 0.00155 | 0.001241 | -0.00306 | -0.0023 |
| 24 | 0.016774 | 0.003822 | 0.156476 | -0.0043 | -0.05478 | -0.00211 | 0.004258 | -0.00021 | -0.00031 | -2.60E-05 |
| 25 | 0.016222 | 0.003059 | 0.153137 | -0.00294 | -0.04946 | 0.000206 | 0.001946 | -0.00092 | 0.000939 | -0.00111 |
| 26 | 0.016883 | 0.003736 | 0.154683 | -0.00474 | -0.05449 | -0.00184 | 0.001608 | -0.00142 | 0.000553 | -0.00246 |
| 27 | 0.017478 | 0.003457 | 0.157136 | -0.00287 | -0.05742 | -0.00098 | -0.00035 | -0.00058 | -0.00029 | 0.000117 |
| 28 | 0.017369 | 0.003266 | 0.155337 | -0.00471 | -0.05358 | -0.00172 | -9.33E-06 | -0.00114 | -7.35E-06 | -0.00042 |
| 29 | 0.016571 | 0.002946 | 0.155473 | -0.00439 | -0.05405 | -0.00078 | 0.001218 | -0.00259 | 0.002103 | -0.00122 |
| 30 | 0.018146 | 0.003418 | 0.15944 | -0.0037 | -0.05897 | -0.00128 | -0.00088 | -0.00129 | -0.00177 | 0.001267 |
| 31 | 0.016663 | 0.003332 | 0.152561 | -0.00388 | -0.04855 | 0.000359 | 0.000504 | -0.00039 | 0.003235 | 0.003653 |
| 32 | 0.017566 | 0.002909 | 0.154957 | -0.00414 | -0.05312 | -0.00183 | 0.000331 | 4.42E-05 | -0.00032 | 0.000309 |
| 33 | 0.016871 | 0.003412 | 0.156007 | -0.00416 | -0.05389 | -0.0026 | -0.00052 | -0.00198 | 0.000444 | -0.00179 |
| 34 | 0.016866 | 0.003179 | 0.156209 | -0.00324 | -0.05661 | -0.00047 | 0.000663 | 0.000111 | -0.00087 | 0.000579 |
| 35 | -0.05154 | 0.009358 | 0.03953 | 0.005058 | 0.132637 | 0.00096 | -0.00573 | -0.00288 | 0.004666 | -0.00925 |
| 36 | -0.05105 | 0.007902 | 0.04525 | 0.001158 | 0.155848 | 0.001978 | -0.01418 | -0.00637 | 0.011854 | -0.00011 |
| 37 | -0.05122 | 0.009636 | 0.036456 | 0.006443 | 0.115575 | 0.00357 | 0.005187 | -0.00288 | 0.002808 | -9.24E-05 |
| 38 | -0.05169 | 0.010523 | 0.049868 | 0.009812 | 0.160258 | 0.007056 | 0.000265 | 0.00742 | 0.00216 | -0.00046 |
| 39 | -0.05144 | 0.009827 | 0.037389 | 0.009254 | 0.126845 | 0.011419 | -0.00165 | 0.004127 | 0.000458 | -0.00327 |
| 40 | -0.05194 | 0.00995 | 0.048657 | 0.004142 | 0.16368 | 0.010455 | -0.00647 | 0.008661 | 0.000526 | 0.004533 |
| 41 | -0.05151 | 0.009267 | 0.046397 | 0.004788 | 0.1403 | -0.00591 | 0.004427 | 0.006993 | -0.00516 | 0.006263 |
| 42 | -0.05176 | 0.008394 | 0.051679 | 0.003237 | 0.170548 | 0.008501 | 3.15E-06 | -0.01016 | 0.011272 | -0.00225 |
| 43 | -0.05194 | 0.009526 | 0.047111 | 0.007263 | 0.154558 | 0.009907 | 0.01259 | 0.011917 | 0.000738 | -0.00296 |
| 44 | -0.05106 | 0.009557 | 0.039131 | 0.007531 | 0.129594 | 0.005559 | -0.01428 | 0.000537 | 0.008723 | 0.004568 |
| 45 | -0.05155 | 0.010268 | 0.051564 | 0.008537 | 0.161567 | -0.00726 | -0.00665 | 0.002843 | -0.00731 | -0.00083 |
| 46 | -0.05238 | 0.009289 | 0.046122 | 0.004715 | 0.147006 | 0.00792 | 0.000162 | -0.00551 | -0.00114 | -0.00623 |
| 47 | -0.05226 | 0.009141 | 0.054139 | 0.002802 | 0.184438 | 0.009128 | -0.01738 | 0.001536 | 0.013224 | -0.00803 |
| 48 | -0.05214 | 0.009722 | 0.053926 | 0.005664 | 0.181759 | 0.005956 | -0.01834 | -0.00362 | 0.005275 | -0.0184 |
| 49 | -0.05135 | 0.00938 | 0.041879 | 0.004977 | 0.14867 | 0.019162 | -0.00067 | -0.00592 | 0.008565 | -0.00772 |
| 50 | -0.05181 | 0.009528 | 0.05098 | 0.006696 | 0.161896 | 0.001205 | 0.003967 | 0.001721 | -0.00035 | 0.005047 |
| 51 | -0.05061 | 0.008902 | 0.038369 | 0.005277 | 0.124727 | 0.015905 | 0.001149 | 0.003509 | 0.001314 | 0.003548 |
| 52 | -0.0516 | 0.00953 | 0.044227 | 0.005166 | 0.154098 | 0.004101 | -0.01286 | 0.002846 | 0.007588 | -0.01283 |
| 53 | -0.05195 | 0.009556 | 0.051475 | 0.003945 | 0.184646 | 0.004839 | -0.02002 | -0.00117 | 0.013438 | -0.01121 |
| 54 | -0.05138 | 0.008586 | 0.049036 | 0.003136 | 0.151933 | -0.00062 | -0.00203 | -0.00109 | -0.01784 | -0.00253 |
| 55 | -0.05116 | 0.009782 | 0.038586 | 0.005816 | 0.122811 | 0.005224 | 0.002495 | -0.00406 | 0.009083 | -0.00969 |
| 56 | -0.05107 | 0.009043 | 0.048905 | 0.003834 | 0.154313 | 0.010268 | 0.000209 | 0.003634 | -0.00652 | -0.00374 |
| 57 | -0.05179 | 0.008148 | 0.05101 | 0.000964 | 0.169671 | 0.003413 | -0.01405 | 0.002642 | 0.005676 | 0.003027 |
| 58 | -0.05136 | 0.009276 | 0.050663 | 0.005049 | 0.169457 | -0.00286 | -0.00704 | -0.01125 | 0.008919 | -0.00033 |
| 59 | -0.05116 | 0.008605 | 0.035877 | 0.008215 | 0.11946 | 0.004213 | 0.010017 | 0.00459 | -0.00602 | 0.000356 |
| 60 | -0.05184 | 0.009443 | 0.047055 | 0.010284 | 0.152636 | 0.003875 | 0.008569 | 0.013319 | -0.0055 | 0.002066 |
| 61 | -0.05145 | 0.009468 | 0.044158 | 0.003783 | 0.143059 | 0.012344 | 0.005998 | -0.00168 | 0.002905 | -0.01273 |
| 62 | -0.05218 | 0.008236 | 0.051141 | 0.002117 | 0.167835 | 0.000519 | 0.002788 | 0.007099 | -0.00463 | -0.00571 |
| 63 | -0.05163 | 0.009218 | 0.048204 | 0.006458 | 0.157287 | 0.005349 | 0.006996 | 0.015567 | 0.010345 | -0.0157 |
| 64 | -0.05114 | 0.008757 | 0.043192 | 0.004385 | 0.13419 | 0.005956 | 0.010791 | 0.003545 | 0.011983 | -0.00092 |
| 65 | -0.05166 | 0.010073 | 0.049077 | 0.008794 | 0.161744 | 0.017361 | 0.0045 | 0.009376 | 0.002768 | 0.00045 |
| 66 | -0.05124 | 0.008691 | 0.039286 | 0.004308 | 0.127662 | 0.01523 | -0.00176 | 0.011549 | -0.00708 | -0.0011 |
| 67 | -0.05177 | 0.009592 | 0.041927 | 0.006578 | 0.145087 | 0.001868 | 0.004237 | -0.00108 | 0.019345 | 0.005627 |
| 68 | 0.048598 | 0.048141 | -0.01781 | -0.01348 | 0.005378 | 0.000264 | -0.00418 | 0.001079 | -0.00178 | 0.004691 |
| 69 | 0.047426 | 0.046598 | -0.01648 | -0.01242 | 0.005901 | 0.001579 | 0.001287 | 0.002999 | 0.000756 | 0.001496 |
| 70 | 0.048263 | 0.047201 | -0.01586 | -0.01262 | 0.004216 | -0.0006 | -0.00261 | 0.001759 | -0.00177 | 0.004563 |
| 71 | 0.047921 | 0.047561 | -0.01617 | -0.01247 | 0.004252 | -5.83E-06 | -0.00267 | -0.00152 | -0.00112 | 0.005236 |
| 72 | 0.047947 | 0.047994 | -0.01756 | -0.01423 | 0.007581 | -0.0035 | -0.0015 | -0.00384 | 0.000482 | -0.00882 |
| 73 | 0.048959 | 0.049545 | -0.01891 | -0.01644 | 0.010313 | -0.00585 | -0.0013 | -0.00846 | -0.00539 | -0.01283 |
| 74 | 0.046548 | 0.045077 | -0.01528 | -0.00997 | 0.003327 | 0.002112 | -0.0021 | -0.00014 | 0.00233 | 0.001809 |
| 75 | 0.047567 | 0.047379 | -0.01659 | -0.01256 | 0.003712 | 0.001965 | 0.000892 | 0.00303 | 0.00152 | 0.002712 |
| 76 | 0.047292 | 0.047555 | -0.01712 | -0.01299 | 0.006427 | 0.00355 | -0.00149 | -0.00105 | 0.011354 | 0.004975 |
| 77 | 0.047459 | 0.047882 | -0.01749 | -0.01373 | 0.004848 | 0.005421 | -0.00016 | -0.00149 | 0.011445 | 0.007785 |
| 78 | 0.048756 | 0.048518 | -0.01706 | -0.01383 | 0.004909 | 0.002774 | 0.002215 | 0.002845 | 0.002542 | 3.21E-05 |
| 79 | 0.048062 | 0.047489 | -0.01711 | -0.01346 | 0.006631 | 0.001229 | 0.0067 | 0.008035 | 0.005535 | 0.008807 |
| 80 | 0.046925 | 0.04689 | -0.01587 | -0.01176 | 0.004034 | 0.00108 | 0.000697 | 0.00187 | 0.002293 | -0.00136 |
| 81 | 0.04819 | 0.048371 | -0.01795 | -0.0148 | 0.006707 | -0.00038 | -0.00429 | -0.00339 | -0.00475 | 0.004957 |
| 82 | 0.048538 | 0.048605 | -0.01714 | -0.01381 | 0.005325 | 0.013392 | -0.0005 | 0.00496 | -0.00654 | 0.00583 |
| 83 | 0.048111 | 0.047238 | -0.0163 | -0.01268 | 0.005063 | -0.00012 | -0.00011 | -0.0025 | 0.002726 | 1.56E-06 |
| 84 | 0.04713 | 0.047331 | -0.01628 | -0.01275 | 0.005748 | -0.00222 | -0.00019 | -0.00115 | -0.00633 | 0.007607 |
| 85 | 0.04957 | 0.049738 | -0.01861 | -0.0169 | 0.009956 | -0.0079 | -0.0033 | -0.00818 | -0.00581 | -0.01567 |
| 86 | 0.048457 | 0.048244 | -0.01799 | -0.01535 | 0.007669 | -0.00224 | -0.00191 | -0.00341 | -0.00047 | -0.00819 |
| 87 | 0.048726 | 0.047557 | -0.01665 | -0.01226 | 0.005587 | 0.00259 | 0.000478 | 0.001705 | 0.002722 | 0.005485 |
| 88 | 0.048097 | 0.048226 | -0.01718 | -0.01226 | 0.004866 | 0.010922 | -0.00024 | 0.003577 | -0.00272 | 0.004278 |
| 89 | 0.04893 | 0.049007 | -0.01825 | -0.01554 | 0.007319 | -0.00672 | 0.00086 | -0.00618 | -0.00539 | -0.01301 |
| 90 | 0.047303 | 0.04611 | -0.01616 | -0.01252 | 0.006068 | 0.001855 | 0.000647 | 0.00113 | 0.001346 | 0.004177 |
| 91 | 0.047999 | 0.046978 | -0.01625 | -0.01258 | 0.005532 | 0.001649 | 0.001529 | -0.0003 | 0.002487 | 0.000805 |
| 92 | 0.047668 | 0.047462 | -0.01714 | -0.01251 | 0.005788 | 0.003834 | -0.00184 | -0.00061 | 0.012502 | 0.009969 |
| 93 | 0.047477 | 0.046616 | -0.01627 | -0.01072 | 0.001617 | 0.001493 | 7.92E-05 | 0.001822 | 0.005079 | 0.004154 |
| 94 | 0.047432 | 0.047761 | -0.01689 | -0.01266 | 0.006145 | 0.004456 | 0.000162 | 0.001799 | 0.013082 | 0.006377 |
| 95 | 0.048553 | 0.047581 | -0.01709 | -0.01329 | 0.005401 | -0.00173 | -0.00103 | -0.00153 | 0.002791 | -0.00065 |
| 96 | 0.047416 | 0.047059 | -0.01632 | -0.01075 | 0.005515 | 0.003296 | 0.001429 | -0.00033 | 0.005582 | 0.005804 |
| 97 | 0.048272 | 0.04784 | -0.01632 | -0.01338 | 0.005172 | 0.000407 | 0.008006 | 0.011428 | 0.002873 | 0.009004 |
| 98 | 0.048168 | 0.048011 | -0.01723 | -0.01333 | 0.006109 | -0.004 | -0.0034 | -0.00086 | -0.00521 | -0.00142 |
| 99 | 0.048984 | 0.048548 | -0.01811 | -0.01529 | 0.008585 | -0.00754 | -0.00417 | -0.01009 | -0.00309 | -0.01539 |
| 100 | 0.048397 | 0.047367 | -0.01133 | -0.01354 | 0.002617 | -0.00583 | -0.00386 | -0.00998 | -0.00395 | -0.01481 |
| 101 | 0.047434 | 0.047405 | -0.0172 | -0.01136 | 0.00661 | 0.005946 | -0.00257 | -0.00198 | 0.012449 | 0.006998 |
| 102 | 0.047631 | 0.047271 | -0.01689 | -0.01212 | 0.00497 | 0.003986 | -0.0004 | -0.00122 | 0.011402 | 0.007134 |
| 103 | 0.049557 | 0.049517 | -0.01827 | -0.01668 | 0.008377 | -0.00445 | 0.000942 | -0.00794 | -0.00394 | -0.01761 |
| 104 | 0.048737 | 0.048455 | -0.01696 | -0.01418 | 0.006284 | -0.00065 | 0.003504 | 0.004832 | -0.00092 | 0.006805 |
| 105 | 0.047842 | 0.047345 | -0.01676 | -0.01225 | 0.0058 | 0.001943 | 0.009539 | 0.015877 | 0.0062 | 0.013411 |
| 106 | 0.048437 | 0.047089 | -0.01647 | -0.01218 | 0.004561 | 0.000697 | 0.000881 | 0.003247 | -0.0003 | 0.001742 |
| 107 | 0.047699 | 0.045792 | -0.01454 | -0.0104 | 0.001887 | 0.002473 | -0.00099 | -0.00059 | -9.33E-06 | -0.00134 |
| 108 | 0.049544 | 0.049297 | -0.01865 | -0.01591 | 0.00879 | -0.0069 | -0.00122 | -0.00849 | -0.00462 | -0.01364 |
| 109 | 0.049552 | 0.049769 | -0.01852 | -0.01658 | 0.008197 | -0.00739 | -0.00126 | -0.00685 | -0.00577 | -0.01367 |
| 110 | 0.049277 | 0.049604 | -0.0193 | -0.01553 | 0.009634 | -0.0096 | -0.00183 | -0.01068 | -0.00203 | -0.01676 |
| 111 | 0.048326 | 0.047298 | -0.01703 | -0.0133 | 0.006473 | -0.00104 | -0.00053 | -0.00155 | 0.004912 | -0.00361 |
| 112 | 0.047639 | 0.047773 | -0.01595 | -0.01298 | 0.005475 | 0.004994 | 0.000427 | 0.00199 | -0.00666 | 0.004909 |
| 113 | 0.049361 | 0.049306 | -0.01818 | -0.0161 | 0.009238 | -0.00614 | 0.000564 | -0.00354 | -0.00511 | -0.01873 |
| 114 | 0.048599 | 0.047959 | -0.01645 | -0.01311 | 0.005129 | -0.00031 | -0.00318 | -0.00016 | -0.00603 | 0.00502 |
| 115 | 0.048163 | 0.04805 | -0.01754 | -0.01281 | 0.006083 | 0.004642 | -0.0045 | -0.00341 | 0.011708 | 0.007454 |
| 116 | 0.049436 | 0.048966 | -0.01703 | -0.0149 | 0.008079 | -0.00197 | 0.004561 | 0.004853 | 0.001667 | 0.007778 |
| 117 | 0.049578 | 0.049454 | -0.01799 | -0.01669 | 0.008089 | -0.00374 | 0.00132 | -0.00266 | -0.00949 | -0.01454 |
| 118 | 0.0471 | 0.046798 | -0.01624 | -0.01095 | 0.003391 | 0.002682 | -0.00326 | -0.00324 | 0.01223 | 0.004369 |
| 119 | 0.048236 | 0.047977 | -0.01664 | -0.01304 | 0.004397 | 0.004945 | -0.00101 | 0.001317 | -0.00285 | 0.008075 |
| 120 | 0.049146 | 0.048622 | -0.01744 | -0.01537 | 0.007551 | -0.00249 | -0.0006 | 0.00081 | 0.000286 | -0.0052 |
| 121 | 0.04891 | 0.049111 | -0.01753 | -0.01507 | 0.008516 | -0.00214 | -0.00078 | -0.0027 | 0.004409 | -0.00919 |
| 122 | 0.049041 | 0.048652 | -0.01766 | -0.01501 | 0.007009 | -0.00395 | -0.00048 | -0.00491 | 0.001735 | -0.01 |
| 123 | 0.043861 | 0.034561 | -0.01674 | 0.004474 | 0.005359 | -0.00233 | -0.00219 | 0.001564 | 0.003957 | 0.006417 |
| 124 | 0.048408 | 0.048021 | -0.01775 | -0.0132 | 0.005039 | 0.017638 | 0.001213 | 0.004548 | -0.00355 | 0.004052 |
| 125 | 0.045731 | 0.043738 | -0.01016 | -0.00775 | -0.00181 | 0.002592 | 0.000274 | -0.00077 | 0.001159 | 0.003092 |
| 126 | 0.048605 | 0.048239 | -0.0174 | -0.01307 | 0.005825 | -0.00519 | -0.00338 | 0.000176 | -0.00277 | 0.003203 |
| 127 | 0.048455 | 0.048609 | -0.01715 | -0.0138 | 0.005825 | -0.0035 | -0.00093 | -0.00134 | -0.00189 | -7.25E-05 |
| 128 | 0.048779 | 0.048372 | -0.01758 | -0.01374 | 0.006179 | 0.013723 | 0.001691 | 0.003014 | -0.0049 | 0.002828 |
| 129 | 0.043515 | 0.035609 | -0.01654 | 0.003923 | 0.003716 | 0.001004 | -0.00059 | 0.003847 | 0.00368 | 0.006314 |
| 130 | 0.049606 | 0.049713 | -0.01971 | -0.01699 | 0.011198 | -0.00675 | -0.00454 | -0.00755 | -0.00588 | -0.01653 |
| 131 | 0.048612 | 0.048627 | -0.01774 | -0.01445 | 0.008984 | -0.00306 | 0.004067 | 0.004249 | 0.00279 | 0.003505 |
| 132 | 0.049536 | 0.049834 | -0.01873 | -0.01737 | 0.009975 | -0.00821 | 0.004951 | 0.000245 | -0.00461 | -0.01356 |
| 133 | 0.04826 | 0.047984 | -0.01654 | -0.01252 | 0.006557 | 0.000681 | 0.009425 | 0.01262 | 0.003426 | 0.011294 |
| 134 | 0.049637 | 0.049033 | -0.01757 | -0.01711 | 0.008012 | -0.00337 | 0.000535 | -0.00171 | -0.00381 | -0.01617 |
| 135 | 0.049237 | 0.048346 | -0.01652 | -0.01438 | 0.006306 | -0.00308 | 0.006249 | 0.011789 | 0.003036 | 0.014067 |
| 136 | 0.049013 | 0.049187 | -0.01703 | -0.01482 | 0.006188 | 0.012035 | 0.002853 | 0.004889 | -0.00608 | 0.006289 |
| 137 | 0.048058 | 0.04773 | -0.01672 | -0.0121 | 0.006567 | 0.002796 | 0.006848 | 0.007759 | 0.004022 | 0.009238 |
| 138 | 0.049567 | 0.048818 | -0.01778 | -0.01446 | 0.008861 | -0.00127 | 0.004285 | 0.003478 | 0.003297 | 0.000168 |
| 139 | 0.048947 | 0.048701 | -0.01834 | -0.0159 | 0.008328 | -0.00373 | 0.002005 | -0.00576 | -0.00559 | -0.01429 |
| 140 | 0.047236 | 0.048271 | -0.01889 | -0.01352 | 0.008263 | 0.002443 | -0.004 | -0.00397 | -0.00173 | 0.009418 |
| 141 | 0.048271 | 0.048813 | -0.01824 | -0.01567 | 0.008673 | 0.001072 | -0.00202 | -0.0063 | 0.010868 | -0.00557 |
| 142 | 0.049739 | 0.049031 | -0.01757 | -0.01564 | 0.009255 | -0.00629 | -0.00152 | -0.00223 | -0.00402 | -0.01803 |
| 143 | 0.047784 | 0.047672 | -0.0173 | -0.01299 | 0.004555 | 0.004039 | -0.00279 | -0.00389 | 0.014189 | 0.005871 |
| 144 | 0.048768 | 0.049536 | -0.01805 | -0.01526 | 0.007081 | 0.001711 | 0.002688 | 0.001728 | -0.00562 | -0.00346 |
| 145 | 0.047599 | 0.046265 | -0.01588 | -0.01148 | 0.003378 | 7.35E-05 | 0.000479 | 0.000209 | 0.000709 | 0.001129 |
| 146 | 0.048441 | 0.048219 | -0.0168 | -0.01324 | 0.003986 | 0.01338 | 0.001726 | 0.005278 | -0.00488 | 0.004412 |
| 147 | 0.049016 | 0.04941 | -0.0186 | -0.01564 | 0.00906 | -0.00559 | -0.00179 | -0.00601 | -0.00276 | -0.01314 |
| 148 | 0.048275 | 0.047622 | -0.01694 | -0.01152 | 0.005564 | -0.00102 | 0.010397 | 0.011557 | 0.004643 | 0.010478 |
| 149 | 0.048195 | 0.047411 | -0.01578 | -0.01298 | 0.004605 | 0.003086 | 0.001244 | 0.001392 | 0.001094 | -8.01E-05 |
| 150 | 0.047462 | 0.047963 | -0.01774 | -0.01271 | 0.00743 | -0.00116 | -0.00398 | -0.00234 | -0.0043 | 0.007512 |
| 151 | 0.047867 | 0.047505 | -0.01631 | -0.01333 | 0.005639 | -0.00199 | 6.37E-05 | 0.003191 | 0.000278 | 0.001619 |
| 152 | 0.048036 | 0.046604 | -0.01583 | -0.0119 | 0.004113 | 0.003341 | 0.000253 | 0.002485 | -0.00055 | 0.000737 |
| 153 | 0.048392 | 0.047714 | -0.01709 | -0.01377 | 0.005968 | 9.23E-05 | 0.007788 | 0.008792 | 0.003982 | 0.011794 |
| 154 | 0.047927 | 0.047354 | -0.017 | -0.01119 | 0.004613 | 0.009572 | 0.005077 | 0.00698 | -0.00404 | 0.000126 |
| 155 | 0.048809 | 0.047651 | -0.01611 | -0.01288 | 0.006137 | 2.02E-05 | 0.009046 | 0.012677 | 0.00347 | 0.009288 |
| 156 | 0.048708 | 0.049096 | -0.01789 | -0.01491 | 0.008609 | -0.00829 | -0.00322 | -0.00834 | -0.0044 | -0.01622 |
| 157 | 0.04826 | 0.047663 | -0.01605 | -0.01307 | 0.003011 | 0.006823 | 0.003912 | 0.005367 | -0.0011 | 0.001517 |
| 158 | 0.047805 | 0.047491 | -0.01651 | -0.01301 | 0.005702 | 0.000142 | -0.00201 | -0.00233 | -0.00703 | 0.00547 |
| 159 | 0.04846 | 0.048288 | -0.01664 | -0.01398 | 0.00649 | -0.00215 | 0.000479 | -0.00298 | 0.00394 | -0.00637 |
| 160 | 0.047883 | 0.04652 | -0.01519 | -0.01155 | 0.003808 | 0.001223 | -0.00188 | -0.00048 | -0.00096 | 0.002583 |
| 161 | 0.048573 | 0.048784 | -0.01713 | -0.01397 | 0.006489 | -0.00152 | 0.000651 | 0.004168 | 3.61E-05 | 0.006915 |
| 162 | 0.048156 | 0.048149 | -0.01676 | -0.01426 | 0.005963 | -0.00033 | -0.00277 | -0.0002 | 0.002731 | 0.0003 |
| 163 | 0.048394 | 0.04823 | -0.01683 | -0.0144 | 0.006775 | 0.0007 | 0.000308 | 0.00041 | 0.007795 | 0.000232 |
| 164 | 0.047447 | 0.047641 | -0.01728 | -0.01387 | 0.005498 | 0.004293 | -0.00606 | -0.0062 | 0.012012 | 0.007609 |
| 165 | 0.04843 | 0.047919 | -0.01722 | -0.01323 | 0.006223 | 0.002856 | -0.00089 | -0.00161 | 0.014159 | 0.009414 |
| 166 | 0.048285 | 0.048057 | -0.01713 | -0.0135 | 0.004765 | -8.07E-05 | 0.004339 | 0.008071 | 0.003095 | 0.007006 |
| 167 | 0.049406 | 0.049365 | -0.01874 | -0.01598 | 0.007323 | 0.003951 | -0.00049 | -0.00159 | -0.00543 | -0.00257 |
| 168 | 0.048366 | 0.048033 | -0.01648 | -0.01406 | 0.006067 | 0.002291 | -0.00388 | 0.000576 | 0.001189 | 0.005865 |
| 169 | 0.047559 | 0.046474 | -0.0146 | -0.01246 | 0.003254 | -0.00132 | 0.000787 | 0.000726 | -0.00385 | 0.011157 |
| 170 | 0.048009 | 0.046484 | -0.01734 | -0.01181 | 0.00786 | -0.00465 | -0.0024 | -0.00212 | -0.00355 | -0.01077 |
| 171 | 0.049154 | 0.048867 | -0.01754 | -0.01624 | 0.008393 | -0.00242 | 0.001162 | -0.00094 | -0.00884 | -0.01249 |
| 172 | 0.049416 | 0.049247 | -0.01847 | -0.01692 | 0.009088 | -0.00637 | -0.00317 | -0.00544 | -0.00636 | -0.01662 |
| 173 | 0.049984 | 0.049175 | -0.01763 | -0.0152 | 0.007293 | -0.00463 | -0.0022 | -0.00333 | -0.0071 | -0.01111 |
| 174 | 0.047501 | 0.048151 | -0.0173 | -0.0131 | 0.006952 | -0.00095 | -0.00642 | -0.00397 | -0.00619 | 0.007519 |
| 175 | 0.048156 | 0.048412 | -0.0181 | -0.01308 | 0.005403 | 0.0079 | -0.00276 | -0.00203 | 0.009475 | 0.004999 |
| 176 | 0.047629 | 0.048199 | -0.0179 | -0.0134 | 0.006129 | -0.00107 | 0.004455 | 0.008707 | 0.000463 | 0.012954 |
| 177 | 0.048552 | 0.047987 | -0.01613 | -0.01355 | 0.005846 | -0.00027 | 0.000344 | 3.81E-05 | 0.000934 | -0.00027 |
| 178 | 0.048374 | 0.047607 | -0.01557 | -0.0114 | 0.00349 | 0.005368 | 0.003721 | 0.008177 | 0.000713 | 0.009467 |
| 179 | 0.048595 | 0.048116 | -0.01629 | -0.01389 | 0.004054 | -0.00046 | -0.00238 | 0.00088 | -0.00342 | 0.004535 |
| 180 | 0.049293 | 0.047883 | -0.01611 | -0.01386 | 0.005088 | 0.001974 | -0.001 | 0.003064 | -0.00356 | 0.003378 |
| 181 | 0.048428 | 0.048005 | -0.01652 | -0.01496 | 0.007049 | 0.002956 | -0.00062 | 0.001001 | -0.00637 | 0.009814 |
| 182 | 0.049124 | 0.048377 | -0.01787 | -0.01511 | 0.008778 | -0.00503 | -0.00172 | -0.00263 | -0.00827 | -0.01603 |
| 183 | 0.049178 | 0.048892 | -0.01732 | -0.01567 | 0.005733 | -0.00018 | -0.00096 | 0.003128 | -0.00097 | 0.008744 |
| 184 | 0.048612 | 0.048067 | -0.0174 | -0.01478 | 0.007683 | 0.000572 | -0.00226 | -0.00498 | 0.006345 | -0.00423 |
| 185 | 0.049655 | 0.049887 | -0.01908 | -0.01632 | 0.008628 | -0.00821 | -0.00061 | -0.01042 | -0.00459 | -0.01985 |
| 186 | 0.048802 | 0.048096 | -0.01714 | -0.01464 | 0.005438 | -0.00035 | 0.003063 | 0.006333 | 0.002263 | 0.010563 |
| 187 | 0.049444 | 0.048881 | -0.01749 | -0.01653 | 0.007384 | -0.00669 | -0.00164 | -0.00431 | -0.00344 | -0.01853 |
| 188 | 0.048154 | 0.047051 | -0.01644 | -0.01217 | 0.004918 | 3.78E-05 | -0.00189 | 0.000647 | 0.000452 | 0.005676 |
| 189 | 0.050098 | 0.049416 | -0.01798 | -0.01765 | 0.009296 | -0.00436 | 0.003783 | 0.002544 | -0.00222 | -0.01303 |
| 190 | 0.047859 | 0.04812 | -0.01735 | -0.01262 | 0.006179 | 0.005423 | -0.00203 | -0.00426 | 0.013547 | 0.003403 |
| 191 | 0.049237 | 0.049329 | -0.01833 | -0.01778 | 0.009948 | -0.00594 | -0.00011 | -0.00455 | -0.00436 | -0.01883 |
| 192 | 0.048766 | 0.048106 | -0.01693 | -0.01232 | 0.004218 | 0.000836 | -0.00311 | 0.000204 | -0.00433 | 0.003349 |
| 193 | 0.047894 | 0.048134 | -0.01726 | -0.01479 | 0.007507 | 0.005103 | -0.00519 | -0.00467 | 0.010052 | 0.00727 |
| 194 | 0.048055 | 0.048174 | -0.01713 | -0.01278 | 0.00614 | 0.001536 | 0.007532 | 0.011579 | 0.00446 | 0.006763 |
| 195 | 0.049229 | 0.048917 | -0.01755 | -0.01651 | 0.009434 | -0.00145 | -0.00042 | -0.00453 | 0.003825 | -0.00865 |
| 196 | 0.048133 | 0.047306 | -0.01662 | -0.01413 | 0.006071 | 9.81E-06 | -0.00262 | 0.002311 | -0.00166 | 0.008917 |
| 197 | 0.048676 | 0.049224 | -0.01785 | -0.01625 | 0.009094 | -0.00628 | -0.00341 | -0.00813 | -0.00487 | -0.015 |
| 198 | 0.048658 | 0.048631 | -0.01769 | -0.0138 | 0.004376 | 0.016606 | 0.001245 | 0.005697 | -0.00517 | 0.006613 |
| 199 | 0.049054 | 0.047689 | -0.01631 | -0.01328 | 0.004084 | 0.016716 | 0.001202 | 0.005467 | -0.0063 | 0.004359 |
| 200 | 0.049462 | 0.049207 | -0.01793 | -0.0149 | 0.007776 | -0.00058 | 0.005183 | 0.004179 | 0.00218 | 0.000282 |
| 201 | 0.047327 | 0.047659 | -0.01739 | -0.01387 | 0.008447 | -0.00133 | -0.00452 | -0.00228 | -0.00566 | 0.007923 |
| 202 | 0.048728 | 0.048986 | -0.01745 | -0.01556 | 0.008917 | -0.00741 | -0.00058 | -0.00709 | -0.00419 | -0.01359 |
| 203 | 0.0468 | 0.045338 | -0.01395 | -0.00899 | 0.001537 | 0.003265 | 0.000128 | 0.002821 | -0.00057 | 0.001996 |
| 204 | 0.048018 | 0.046831 | -0.01627 | -0.0126 | 0.004154 | 0.002858 | 0.00123 | 0.005948 | -0.00025 | 0.009504 |
| 205 | 0.048997 | 0.048852 | -0.01668 | -0.01446 | 0.00591 | 0.014766 | 0.002539 | 0.003739 | -0.00559 | 0.00516 |
| 206 | 0.047692 | 0.04633 | -0.01541 | -0.01154 | 0.005076 | 0.001911 | 0.001389 | 0.00623 | 0.000942 | 0.007608 |
| 207 | 0.049098 | 0.049541 | -0.0185 | -0.01539 | 0.009186 | -0.00771 | -0.00558 | -0.00721 | -0.00325 | -0.015 |
| 208 | 0.046851 | 0.045809 | -0.01538 | -0.01091 | 0.0037 | 0.002071 | 0.000957 | 0.003255 | -0.00125 | 0.007089 |
| 209 | -0.0486 | 0.024093 | -0.01647 | 0.064368 | -0.03168 | 0.055483 | 0.038078 | 0.032588 | -0.07663 | 0.010854 |
| 210 | -0.04874 | 0.021928 | -0.01266 | 0.054097 | -0.01923 | 0.067422 | 0.0028 | 0.028493 | -0.03597 | 0.061746 |
| 211 | -0.04903 | 0.02288 | -0.01715 | 0.058891 | -0.02751 | -0.03912 | -0.05308 | -0.06945 | 0.11169 | -0.1391 |
| 212 | -0.04883 | 0.023732 | -0.01789 | 0.060833 | -0.03381 | -0.10212 | 0.041551 | 0.016816 | -0.04955 | -0.15986 |
| 213 | -0.04883 | 0.025575 | -0.01922 | 0.069897 | -0.04848 | 0.244773 | -0.02783 | -0.02267 | -0.07713 | -0.01421 |
| 214 | -0.04802 | 0.023838 | -0.01619 | 0.062222 | -0.02721 | 0.067427 | 0.022286 | 0.029877 | -0.03249 | 0.02565 |
| 215 | -0.04861 | 0.025449 | -0.01719 | 0.070685 | -0.04049 | 0.248263 | -0.02859 | -0.01758 | -0.09457 | -0.00882 |
| 216 | -0.04851 | 0.023079 | -0.01652 | 0.059021 | -0.03245 | -0.12732 | -0.00093 | -0.01772 | -0.05043 | -0.21712 |
| 217 | -0.04827 | 0.022618 | -0.01857 | 0.059736 | -0.03948 | 0.138755 | -0.13024 | -0.14084 | 0.129605 | 0.05942 |
| 218 | -0.04899 | 0.024063 | -0.01521 | 0.060518 | -0.02683 | -0.03801 | 0.079156 | 0.08047 | 0.029489 | 0.112448 |
| 219 | -0.04848 | 0.022929 | -0.01656 | 0.061381 | -0.03588 | 0.133809 | -0.10869 | -0.12464 | 0.119262 | 0.016136 |
| 220 | -0.04893 | 0.022064 | -0.01777 | 0.056281 | -0.03168 | -0.01363 | -0.04548 | -0.07465 | 0.173268 | -0.18718 |
| 221 | -0.04873 | 0.018909 | -0.01062 | 0.043662 | -0.00646 | -0.01214 | -0.0333 | -0.0123 | -0.05221 | 0.058832 |
| 222 | -0.04912 | 0.024686 | -0.01717 | 0.067665 | -0.03564 | 0.068535 | 0.145833 | 0.113614 | -0.01479 | -0.04969 |
| 223 | -0.04921 | 0.025714 | -0.01786 | 0.069248 | -0.03691 | -0.04106 | 0.004864 | 0.014405 | -0.06003 | -0.13031 |
| 224 | -0.04847 | 0.017061 | -0.00709 | 0.034066 | 5.77E-05 | -0.01636 | 0.033257 | 0.026181 | 0.01497 | 0.018784 |
| 225 | -0.04847 | 0.020994 | -0.01097 | 0.050076 | -0.00798 | -0.03124 | 0.114977 | 0.098349 | 0.076584 | 0.085072 |
| 226 | -0.04892 | 0.020415 | -0.014 | 0.048214 | -0.01187 | -0.03043 | -0.04159 | -0.00879 | -0.08254 | 0.017573 |
| 227 | -0.04807 | 0.024251 | -0.01761 | 0.063768 | -0.04052 | 0.224451 | -0.0156 | -0.00669 | -0.12698 | -0.01791 |
| 228 | -0.04818 | 0.021831 | -0.01621 | 0.05348 | -0.03216 | 0.027324 | -0.12101 | -0.14336 | 0.206225 | 0.057118 |
| 229 | -0.0482 | 0.023137 | -0.01569 | 0.058249 | -0.02174 | -0.12336 | -0.08386 | -0.04491 | -0.14675 | 0.047509 |
| 230 | -0.04839 | 0.02372 | -0.0169 | 0.061575 | -0.03532 | -0.09496 | -0.11598 | -0.07764 | -0.08458 | 0.071811 |
| 231 | -0.04891 | 0.025112 | -0.01669 | 0.066365 | -0.02537 | -0.04158 | 0.133929 | 0.112701 | 0.0728 | 0.062759 |
| 232 | -0.04884 | 0.025227 | -0.01902 | 0.066638 | -0.03911 | 0.091399 | -0.0274 | -0.02633 | 0.019057 | -0.17511 |
| 233 | -0.04857 | 0.023407 | -0.01944 | 0.06593 | -0.04325 | 0.106061 | 0.100111 | 0.076495 | -0.01508 | -0.0364 |
| 234 | -0.04809 | 0.019445 | -0.01052 | 0.043139 | -0.01095 | -0.04559 | -0.06484 | -0.02763 | -0.13855 | 0.106679 |
| 235 | -0.04882 | 0.023798 | -0.01636 | 0.058685 | -0.02986 | -0.0912 | 0.001205 | -0.0086 | -0.05768 | -0.10615 |
| 236 | -0.04807 | 0.018662 | -0.00908 | 0.041402 | -0.0023 | -0.08433 | -0.06212 | -0.02441 | -0.1293 | 0.092276 |
| 237 | -0.04889 | 0.023855 | -0.01654 | 0.065315 | -0.03029 | 0.115312 | -0.04678 | -0.00281 | -0.13232 | -0.00017 |
| 238 | -0.04867 | 0.024242 | -0.01763 | 0.063405 | -0.03766 | -0.01108 | -0.11744 | -0.14152 | 0.189179 | 0.038072 |
| 239 | -0.04784 | 0.022522 | -0.01827 | 0.056517 | -0.03781 | 0.015684 | -0.08997 | -0.11332 | 0.186283 | 0.092197 |
| 240 | -0.04807 | 0.025358 | -0.01937 | 0.068121 | -0.04586 | 0.215766 | -0.02831 | -0.01224 | -0.10394 | -0.02203 |
| 241 | -0.04793 | 0.028128 | -0.02045 | 0.077577 | -0.04901 | 0.189436 | 0.057509 | 0.062485 | -0.05909 | 0.046374 |
| 242 | -0.04762 | 0.022218 | -0.01469 | 0.054144 | -0.01651 | -0.11498 | 0.014667 | 0.038922 | -0.03226 | 0.14733 |
| 243 | -0.04892 | 0.024175 | -0.01659 | 0.060919 | -0.03256 | -0.05592 | -0.00191 | -0.01395 | -0.05582 | -0.11012 |
| 244 | -0.0485 | 0.02294 | -0.01661 | 0.056776 | -0.02908 | 0.006092 | -0.08423 | -0.12747 | 0.183966 | 0.037893 |
| 245 | -0.04795 | 0.021153 | -0.01181 | 0.05127 | -0.01113 | 0.065687 | -0.012 | 0.000913 | -0.02815 | 0.067186 |
| 246 | -0.04892 | 0.023386 | -0.01762 | 0.063051 | -0.03626 | 0.059098 | 0.137738 | 0.114039 | 0.020988 | -0.07966 |
| 247 | -0.0484 | 0.02289 | -0.01228 | 0.058093 | -0.0166 | -0.04165 | 0.132991 | 0.11196 | 0.096243 | 0.09626 |
| 248 | -0.04884 | 0.020577 | -0.01612 | 0.050582 | -0.02977 | 0.096187 | 0.100201 | 0.084475 | 0.000688 | -0.07618 |
| 249 | -0.04847 | 0.025191 | -0.01739 | 0.060784 | -0.02708 | -0.12357 | -0.00601 | -0.01857 | -0.09319 | -0.17517 |
| 250 | -0.04815 | 0.022799 | -0.0181 | 0.058198 | -0.03859 | -0.01039 | -0.14122 | -0.15291 | 0.169495 | 0.047041 |
| 251 | -0.04885 | 0.020809 | -0.01087 | 0.051039 | -0.00852 | -0.01787 | -0.01822 | -0.02435 | 0.098949 | -0.20273 |
| 252 | -0.04901 | 0.025594 | -0.02025 | 0.071335 | -0.04929 | 0.22594 | -0.03367 | -0.03878 | -0.05417 | -0.00564 |
| 253 | -0.04891 | 0.020063 | -0.01448 | 0.049612 | -0.01734 | -0.00717 | -0.01726 | -0.01963 | 0.108958 | -0.19132 |
| 254 | -0.04873 | 0.022039 | -0.01333 | 0.056493 | -0.01759 | -0.01438 | -0.06052 | -0.04289 | -0.01369 | 0.033067 |
| 255 | -0.04843 | 0.024549 | -0.01738 | 0.065733 | -0.03237 | -0.14492 | 0.057928 | 0.033083 | -0.03034 | -0.11785 |
| 256 | -0.04826 | 0.020695 | -0.0138 | 0.048707 | -0.0236 | 0.017543 | -0.01388 | -0.00739 | -0.00084 | 0.006046 |
| 257 | -0.04805 | 0.024438 | -0.01982 | 0.068238 | -0.046 | 0.079893 | 0.074148 | 0.068292 | -0.00222 | -0.07125 |
| 258 | -0.04937 | 0.02187 | -0.01334 | 0.054122 | -0.02471 | -0.01249 | 0.042249 | 0.042918 | 0.001892 | -0.02043 |
| 259 | -0.0488 | 0.022317 | -0.01432 | 0.054035 | -0.02423 | -0.04012 | 0.010415 | -0.02048 | 0.031759 | -0.00359 |
| 260 | -0.04801 | 0.025961 | -0.01743 | 0.069647 | -0.03771 | 0.228883 | -0.01512 | -0.006 | -0.09992 | -0.02816 |
| 261 | -0.04844 | 0.021206 | -0.01507 | 0.055939 | -0.02374 | 0.145971 | -0.05255 | -0.03776 | -0.04793 | 0.033249 |
| 262 | -0.04908 | 0.017291 | -0.00811 | 0.037298 | -0.0092 | 0.230256 | -0.0089 | -0.02136 | -0.11532 | -0.01443 |
| 263 | -0.04836 | 0.020548 | -0.01516 | 0.049127 | -0.02202 | -0.05539 | -0.01948 | 0.00338 | -0.00402 | 0.071971 |
| 264 | -0.04796 | 0.019289 | -0.01203 | 0.046325 | -0.0201 | 0.03416 | -0.10476 | -0.13577 | 0.18628 | 0.068719 |
| 265 | -0.04882 | 0.023185 | -0.01536 | 0.059132 | -0.02436 | -0.05035 | 0.065358 | 0.062889 | 0.03637 | 0.099067 |
| 266 | -0.04829 | 0.025088 | -0.01837 | 0.065966 | -0.03636 | -0.05454 | 0.050851 | 0.053604 | 0.047587 | 0.136736 |
| 267 | -0.04934 | 0.023858 | -0.01833 | 0.065901 | -0.03659 | 0.08698 | 0.113281 | 0.096909 | 0.004965 | -0.04664 |
| 268 | -0.0484 | 0.021655 | -0.01588 | 0.054347 | -0.03203 | 0.000867 | 0.001139 | -0.00643 | 0.025603 | -0.01242 |
| 269 | -0.04813 | 0.022121 | -0.01548 | 0.05731 | -0.02246 | -0.09242 | -0.103 | -0.05024 | -0.19156 | 0.149932 |
| 270 | -0.04877 | 0.021391 | -0.01669 | 0.051257 | -0.02415 | -0.0255 | 0.017519 | 0.015279 | 0.032929 | 0.014225 |
| 271 | -0.0488 | 0.025416 | -0.0187 | 0.064287 | -0.03359 | -0.10215 | -0.00053 | -0.01413 | -0.07612 | -0.16472 |
| 272 | -0.04778 | 0.022291 | -0.01437 | 0.055219 | -0.02023 | -0.09836 | -0.06917 | -0.02359 | -0.17572 | 0.110966 |
| 273 | -0.04945 | 0.021114 | -0.01293 | 0.051593 | -0.01819 | -0.02235 | 0.016921 | 0.031248 | -0.06741 | 0.016492 |
| 274 | -0.04794 | 0.019584 | -0.0123 | 0.046531 | -0.01655 | -0.0003 | -0.00055 | 0.008691 | 0.001348 | -0.01488 |
| 275 | -0.04821 | 0.022575 | -0.01665 | 0.058259 | -0.03254 | -0.00122 | -0.02455 | -0.00767 | 0.012327 | -0.00534 |
| 276 | -0.04871 | 0.020867 | -0.01507 | 0.052403 | -0.02366 | -0.02995 | -0.01504 | 0.004931 | 0.007054 | 0.018834 |
| 277 | -0.04907 | 0.020668 | -0.01364 | 0.047 | -0.01717 | -0.02611 | 0.015066 | 0.017822 | 0.033927 | -0.0048 |
| 278 | -0.04853 | 0.023106 | -0.01646 | 0.059357 | -0.02819 | -0.02186 | -0.01271 | -0.00647 | 0.084769 | -0.17148 |
| 279 | -0.04802 | 0.024234 | -0.01706 | 0.05966 | -0.0305 | -0.10368 | 0.062327 | 0.05337 | 0.030224 | 0.067769 |
| 280 | -0.04881 | 0.023313 | -0.01603 | 0.059891 | -0.02446 | -0.06657 | 0.105051 | 0.095317 | 0.082597 | 0.101072 |
| 281 | -0.04788 | 0.021038 | -0.01343 | 0.050206 | -0.02225 | -0.04443 | 0.084808 | 0.085003 | 0.068456 | 0.110013 |
| 282 | -0.04835 | 0.021493 | -0.0176 | 0.052462 | -0.0362 | 0.035399 | -0.08701 | -0.09532 | 0.100597 | 0.038659 |
| 283 | -0.0484 | 0.02335 | -0.01747 | 0.059021 | -0.03284 | 0.016188 | -0.11168 | -0.13257 | 0.184985 | 0.047431 |
| 284 | -0.04902 | 0.022991 | -0.01754 | 0.057324 | -0.03153 | -0.08212 | -0.05293 | -0.05058 | 0.028207 | -0.01157 |
| 285 | -0.04915 | 0.021932 | -0.01563 | 0.056031 | -0.02118 | -0.02874 | -0.05657 | -0.01277 | -0.06597 | 0.024198 |
| 286 | -0.04846 | 0.019734 | -0.01149 | 0.047467 | -0.00577 | -0.01677 | -0.03661 | 0.003049 | -0.06745 | 0.017391 |
| 287 | -0.04816 | 0.024452 | -0.01817 | 0.064168 | -0.03221 | -0.16727 | -0.14138 | -0.07034 | -0.21671 | 0.115529 |
| 288 | -0.0483 | 0.022105 | -0.01681 | 0.05172 | -0.02608 | -0.0329 | -0.00301 | 0.005638 | -0.01349 | -0.00435 |
| 289 | -0.04839 | 0.025833 | -0.01978 | 0.068199 | -0.0353 | -0.11584 | -0.04964 | -0.03307 | -0.07662 | 0.001144 |
| 290 | -0.04882 | 0.024844 | -0.01764 | 0.066665 | -0.03257 | -0.16135 | 0.014302 | -0.00158 | -0.06322 | -0.24538 |
| 291 | -0.04778 | 0.023109 | -0.01647 | 0.056434 | -0.02361 | -0.10949 | -0.11897 | -0.06701 | -0.19061 | 0.11695 |
| 292 | -0.04857 | 0.02077 | -0.0116 | 0.046505 | -0.00577 | -0.02194 | 0.018171 | 0.014256 | 0.005865 | 0.020928 |
| 293 | -0.0485 | 0.022459 | -0.01538 | 0.057923 | -0.02391 | -0.02257 | 0.016662 | -0.01722 | 0.02345 | 0.053876 |
| 294 | -0.04776 | 0.021394 | -0.01648 | 0.050734 | -0.02861 | -0.06385 | 0.055761 | 0.043132 | 0.059574 | 0.10543 |
| 295 | -0.04797 | 0.021136 | -0.01359 | 0.04867 | -0.01799 | -0.12126 | -0.01311 | -0.04306 | -0.07798 | -0.16505 |
| 296 | -0.04849 | 0.019739 | -0.01327 | 0.045324 | -0.02176 | -0.01006 | -0.02791 | -0.00178 | -0.01148 | 0.026386 |
| 297 | -0.0491 | 0.022199 | -0.01583 | 0.05867 | -0.02477 | -0.06452 | 0.147315 | 0.135406 | 0.083626 | 0.105913 |
| 298 | -0.04912 | 0.020834 | -0.01297 | 0.052982 | -0.01858 | -0.0878 | 0.010081 | 0.022904 | -0.01138 | -0.11356 |
| 299 | -0.04879 | 0.021928 | -0.01654 | 0.055644 | -0.02788 | -0.00212 | -0.02091 | -0.01009 | 0.036326 | -0.08523 |
| 300 | -0.0492 | 0.022883 | -0.01401 | 0.061734 | -0.02414 | -0.05884 | 0.137061 | 0.134242 | 0.086497 | 0.08177 |
| 301 | -0.0482 | 0.025584 | -0.02026 | 0.069142 | -0.04287 | -0.04993 | 0.105379 | 0.103328 | 0.105665 | 0.14969 |
| 302 | -0.04882 | 0.02092 | -0.01283 | 0.050717 | -0.00916 | -0.03603 | -0.03405 | -0.00476 | -0.04077 | -0.00603 |
| 303 | -0.04821 | 0.018878 | -0.01372 | 0.04103 | -0.01817 | -0.00164 | -0.01014 | -0.00985 | 0.008193 | 0.055395 |
| 304 | -0.04935 | 0.023989 | -0.01782 | 0.064148 | -0.04007 | 0.08566 | 0.131873 | 0.102146 | -0.01967 | -0.06979 |
| 305 | -0.04857 | 0.022647 | -0.01562 | 0.055976 | -0.02522 | -0.03034 | 0.007267 | -0.01375 | 0.003674 | 0.016078 |
| 306 | -0.04857 | 0.02147 | -0.01676 | 0.054103 | -0.02662 | -0.01675 | 0.012595 | -0.01357 | 0.011876 | 0.03158 |
| 307 | -0.04867 | 0.022805 | -0.01563 | 0.060729 | -0.02504 | -0.02157 | 0.028488 | 0.005187 | 0.001655 | 0.071836 |
| 308 | -0.04915 | 0.023771 | -0.01785 | 0.059169 | -0.03097 | -0.08104 | -0.02366 | -0.03227 | 0.073125 | -0.27115 |
| 309 | -0.04832 | 0.024713 | -0.02104 | 0.066298 | -0.04616 | 0.01263 | -0.02104 | -0.04668 | 0.233866 | 0.121578 |
| 310 | -0.04845 | 0.024611 | -0.01685 | 0.062495 | -0.02677 | -0.06348 | 0.06451 | 0.043002 | 0.031792 | 0.012324 |
| 311 | -0.04914 | 0.021549 | -0.01431 | 0.053959 | -0.02126 | -0.00779 | 0.015098 | 0.019342 | 0.036802 | -0.00895 |
| 312 | -0.04836 | 0.022334 | -0.01298 | 0.057676 | -0.02565 | 0.217306 | -0.00605 | 0.005909 | -0.10128 | -0.0155 |
| 313 | -0.04803 | 0.020023 | -0.00788 | 0.048584 | -0.0069 | 0.136351 | -0.03017 | -0.0217 | -0.07805 | 0.0259 |
| 314 | -0.04866 | 0.019607 | -0.00785 | 0.046593 | -0.0015 | -0.04346 | 0.089957 | 0.095532 | 0.057613 | 0.075154 |
| 315 | -0.04849 | 0.025082 | -0.01991 | 0.063753 | -0.03426 | -0.12539 | -0.06655 | -0.04998 | -0.04396 | -0.03893 |
| 316 | -0.04888 | 0.025779 | -0.02017 | 0.072736 | -0.04583 | 0.040833 | 0.199059 | 0.158724 | 0.050171 | -0.00132 |
| 317 | -0.04838 | 0.021492 | -0.01309 | 0.05467 | -0.01612 | -0.10543 | -0.07136 | -0.03138 | -0.19256 | 0.152849 |
| 318 | 0.031606 | -0.07174 | -0.01258 | 0.020443 | 0.004346 | 0.000362 | 0.000998 | -0.00107 | -0.00224 | -0.00089 |
| 319 | 0.031571 | -0.07198 | -0.01235 | 0.021342 | 0.006474 | -2.67E-05 | -0.00011 | 0.00071 | -0.00143 | 0.00037 |
| 320 | 0.031277 | -0.07173 | -0.01301 | 0.020909 | 0.004115 | -0.00172 | 0.004199 | -0.00341 | -0.0003 | 5.63E-05 |
| 321 | 0.031542 | -0.07252 | -0.01252 | 0.021717 | 0.005302 | -8.22E-05 | -1.11E-05 | -5.97E-05 | -1.37E-05 | -0.00013 |
| 322 | 0.031228 | -0.07107 | -0.01195 | 0.019883 | 0.004949 | 0.00135 | 0.003737 | -0.00065 | -0.00137 | -0.00135 |
| 323 | 0.031475 | -0.07043 | -0.01163 | 0.018377 | 0.004465 | -0.0023 | 0.003675 | 0.002761 | -0.00141 | -0.00013 |
| 324 | 0.031283 | -0.07123 | -0.01275 | 0.019061 | 0.007138 | -0.00122 | 0.000994 | 0.000723 | -1.64E-05 | 0.000489 |
| 325 | 0.031058 | -0.07145 | -0.01198 | 0.01886 | 0.006037 | 0.000732 | 0.003128 | -0.00145 | 0.001498 | 0.001962 |
| 326 | 0.031489 | -0.07179 | -0.01141 | 0.020098 | 0.005085 | -0.00058 | 0.000777 | -0.00092 | 0.002069 | 0.001322 |
| 327 | 0.03173 | -0.07276 | -0.0124 | 0.023597 | 0.007769 | 9.25E-05 | 0.000541 | -0.00303 | -0.00133 | -0.00213 |
| 328 | 0.031302 | -0.07091 | -0.01214 | 0.019222 | 0.005517 | -6.36E-06 | 0.001613 | 0.004869 | -0.00087 | -0.00024 |
| 329 | 0.031218 | -0.07183 | -0.01265 | 0.019698 | 0.003246 | -0.0028 | 0.002268 | -0.00026 | -0.00488 | 0.001289 |
| 330 | 0.030997 | -0.0716 | -0.01266 | 0.020368 | 0.006432 | 0.001037 | -0.00059 | -0.00077 | -0.00052 | -0.00269 |
| 331 | 0.031045 | -0.07138 | -0.01226 | 0.019463 | 0.003694 | -0.00123 | 0.002699 | 0.001422 | -0.00258 | -0.00044 |
| 332 | 0.031743 | -0.07297 | -0.01225 | 0.021333 | 0.007085 | 0.000575 | 0.002708 | -0.00438 | 0.000863 | -0.00135 |
| 333 | 0.0316 | -0.0722 | -0.01217 | 0.020787 | 0.005733 | -0.00065 | -0.00058 | 0.001609 | 0.00048 | 0.000256 |
| 334 | 0.031055 | -0.07226 | -0.01266 | 0.01984 | 0.005058 | -0.00052 | -0.00032 | -0.00071 | -0.00141 | 0.000734 |
| 335 | 0.03093 | -0.07209 | -0.01213 | 0.019576 | 0.004903 | 0.000744 | 0.001162 | -0.00041 | -0.00085 | -0.0014 |
| 336 | 0.031192 | -0.07136 | -0.01195 | 0.020044 | 0.005021 | 0.000928 | 0.000741 | -8.21E-05 | 0.000124 | -0.00061 |
| 337 | 0.030615 | -0.07084 | -0.01145 | 0.017514 | 0.002731 | -0.00032 | 0.000393 | 0.000586 | 0.000201 | -0.00295 |
| 338 | 0.030967 | -0.07144 | -0.01317 | 0.021627 | 0.006153 | 9.44E-05 | 0.001154 | -0.00446 | -0.00026 | -0.00074 |
| 339 | 0.030893 | -0.06976 | -0.01193 | 0.016809 | 0.00471 | -0.00015 | 0.000926 | -0.00018 | 0.001298 | 0.000739 |
| 340 | 0.031526 | -0.07207 | -0.01239 | 0.020086 | 0.005168 | -0.00057 | -2.98E-05 | 0.00202 | -0.00116 | 0.00072 |
| 341 | 0.030704 | -0.07171 | -0.0118 | 0.018748 | 0.006 | 0.001142 | 0.000204 | -0.00161 | 0.003599 | 0.001777 |
| 342 | 0.031468 | -0.0712 | -0.01187 | 0.018818 | 0.005951 | -0.00046 | 0.001584 | -0.00202 | 0.000357 | 0.002094 |
| 343 | 0.031717 | -0.07221 | -0.01212 | 0.019773 | 0.006683 | -0.00028 | -0.01156 | 0.008106 | 0.001421 | 0.002468 |
| 344 | 0.030713 | -0.07205 | -0.01166 | 0.019658 | 0.005913 | -0.001 | -0.00131 | 0.001422 | 0.002939 | 0.002215 |
| 345 | 0.031941 | -0.07225 | -0.01233 | 0.020575 | 0.004155 | -0.00056 | 0.000223 | -0.00034 | 0.001472 | 0.00124 |
| 346 | 0.031414 | -0.07126 | -0.01239 | 0.020273 | 0.00545 | 0.000373 | 0.0049 | -0.0034 | -0.00072 | -0.00034 |
| 347 | 0.030045 | -0.07068 | -0.01239 | 0.017899 | 0.004853 | -0.0012 | 0.002841 | -0.00022 | 0.001885 | -0.00144 |
| 348 | 0.031537 | -0.07314 | -0.01226 | 0.023719 | 0.006357 | 0.0013 | 0.001113 | -0.00425 | -0.00033 | -0.0008 |
| 349 | 0.030923 | -0.07159 | -0.01231 | 0.019274 | 0.006513 | -6.44E-05 | 0.000765 | -0.00071 | -0.00262 | 0.000764 |
| 350 | 0.031354 | -0.07119 | -0.01212 | 0.018423 | 0.005483 | 0.000314 | -0.00074 | 0.001596 | 0.002209 | -0.00097 |
| 351 | 0.031313 | -0.07298 | -0.01298 | 0.022015 | 0.006487 | 5.35E-05 | 0.000499 | -0.00334 | 0.000814 | -0.00108 |
| 352 | 0.031615 | -0.07149 | -0.01217 | 0.01921 | 0.004961 | -0.00049 | -0.00201 | -0.00399 | -0.00089 | 0.003676 |
| 353 | 0.030796 | -0.07144 | -0.01255 | 0.019353 | 0.006311 | -0.00139 | -0.00154 | 0.00276 | 0.001752 | -0.0015 |
| 354 | 0.031489 | -0.07164 | -0.0119 | 0.018801 | 0.003729 | 0.000622 | -0.00136 | -0.00267 | -0.00335 | 0.000865 |
| 355 | 0.031373 | -0.07001 | -0.01132 | 0.017964 | 0.002901 | 0.0013 | 0.00168 | -0.00042 | 0.000356 | -0.00193 |
| 356 | 0.031821 | -0.07173 | -0.01177 | 0.019395 | 0.004654 | 0.000734 | 0.000293 | -0.00053 | -0.00121 | 0.002457 |
| 357 | 0.031775 | -0.07241 | -0.01212 | 0.021445 | 0.005861 | -0.00252 | 0.002235 | -0.00236 | 0.001223 | -0.00085 |
| 358 | 0.030894 | -0.07202 | -0.01283 | 0.020445 | 0.004127 | -0.00195 | 0.001582 | -0.00068 | -0.00397 | -0.00158 |
| 359 | 0.030617 | -0.07128 | -0.01205 | 0.019085 | 0.004543 | -0.00076 | 0.000848 | -0.00238 | 0.001668 | -0.00218 |
| 360 | 0.031765 | -0.07227 | -0.01232 | 0.02081 | 0.00528 | -0.00084 | 0.001712 | -0.00366 | -0.00103 | 0.00186 |
| 361 | 0.03154 | -0.0721 | -0.01208 | 0.021146 | 0.003865 | -0.00097 | 0.000202 | 0.002442 | -0.00129 | -5.12E-05 |
| 362 | 0.031362 | -0.07243 | -0.01216 | 0.021836 | 0.004733 | 0.000122 | 0.001811 | -0.0035 | -0.00092 | -0.00018 |
| 363 | 0.030348 | -0.07135 | -0.01301 | 0.018477 | 0.005327 | -0.00253 | 0.001818 | 0.00116 | 0.001573 | 0.002309 |
| 364 | 0.031237 | -0.07153 | -0.01181 | 0.018387 | 0.005469 | 0.000781 | 0.00292 | -0.0018 | 0.001858 | 0.00123 |
| 365 | 0.031808 | -0.07072 | -0.01104 | 0.018436 | 0.003016 | 0.002985 | -0.00086 | -0.00284 | -0.00209 | -0.00111 |
| 366 | 0.031579 | -0.07098 | -0.01173 | 0.018612 | 0.004733 | -0.0003 | 0.000284 | -0.00076 | 0.002062 | -0.00275 |
| 367 | 0.031207 | -0.07085 | -0.01168 | 0.018514 | 0.004299 | 0.00255 | 0.001062 | -0.00174 | 0.001598 | 0.002085 |
| 368 | 0.031631 | -0.07257 | -0.01187 | 0.020352 | 0.005763 | -0.00115 | -0.00824 | 0.008188 | 0.001392 | 0.000512 |
| 369 | 0.031257 | -0.072 | -0.01191 | 0.021927 | 0.006024 | -0.00036 | 0.000703 | -0.00149 | -0.00257 | 0.000998 |
| 370 | 0.031116 | -0.07143 | -0.01205 | 0.018901 | 0.006334 | -0.00071 | 0.000134 | -0.00076 | 0.001409 | -0.00066 |
| 371 | 0.031243 | -0.07215 | -0.01292 | 0.0199 | 0.006944 | -0.00018 | -0.00146 | 0.000463 | 0.000665 | 0.001675 |
| 372 | 0.031202 | -0.07133 | -0.01246 | 0.020369 | 0.00399 | 3.08E-05 | -0.00053 | 0.000918 | 0.003753 | -0.00328 |
| 373 | 0.03096 | -0.07077 | -0.01174 | 0.018859 | 0.004846 | 0.001112 | 0.001031 | 0.001991 | 0.000859 | -0.00193 |
| 374 | 0.031491 | -0.07207 | -0.01278 | 0.021007 | 0.005406 | 0.000548 | 0.001678 | -0.00038 | -0.00011 | 6.04E-07 |
| 375 | 0.030995 | -0.07102 | -0.01075 | 0.019097 | 0.003535 | 0.001806 | -0.00204 | -0.00063 | -0.00194 | 0.000921 |
| 376 | 0.030846 | -0.07157 | -0.01276 | 0.018954 | 0.00393 | -7.67E-05 | 0.000585 | 0.000592 | 0.001826 | -0.00202 |
| 377 | 0.031104 | -0.0713 | -0.01253 | 0.019379 | 0.004672 | -0.00127 | -0.00121 | 0.00384 | 0.001826 | -0.00307 |
| 378 | 0.031435 | -0.07197 | -0.01315 | 0.020201 | 0.006452 | -0.00037 | -0.00169 | 0.00309 | 0.000504 | -0.0008 |
| 379 | 0.031385 | -0.07201 | -0.01244 | 0.021181 | 0.005212 | -0.00046 | 0.002309 | -0.00303 | 0.002564 | -0.00234 |
| 380 | 0.031102 | -0.07106 | -0.0113 | 0.019659 | 0.005151 | 0.000653 | -0.00151 | 0.004164 | -0.00039 | 0.002312 |
| 381 | 0.031793 | -0.07114 | -0.01173 | 0.019354 | 0.005214 | -0.00132 | -0.00057 | 0.000298 | 0.003485 | -0.00132 |
| 382 | 0.031299 | -0.06996 | -0.01148 | 0.016794 | 0.003708 | 0.001212 | -0.00365 | 0.001387 | 0.00319 | -0.00053 |
| 383 | 0.030334 | -0.07126 | -0.01149 | 0.018057 | 0.002941 | 0.000436 | 6.87E-05 | 0.000602 | -0.00241 | 0.00089 |
| 384 | 0.031643 | -0.07202 | -0.01205 | 0.020426 | 0.004309 | 0.000609 | -0.00029 | 0.003732 | -0.00032 | 0.002184 |
| 385 | 0.031803 | -0.0726 | -0.01271 | 0.020399 | 0.005874 | 0.000204 | -0.00828 | 0.009156 | -0.00042 | 0.002069 |
| 386 | 0.030933 | -0.07166 | -0.01217 | 0.018952 | 0.005555 | 0.000576 | -0.00065 | 0.001189 | 0.001715 | 0.000557 |
| 387 | 0.030938 | -0.07102 | -0.01188 | 0.01652 | 0.00388 | 0.00177 | 0.000172 | -0.00066 | 0.003251 | 0.001662 |
| 388 | 0.030661 | -0.07229 | -0.01258 | 0.021297 | 0.004144 | -0.00061 | 0.000662 | -0.00386 | 0.002503 | -0.00241 |
| 389 | 0.032175 | -0.07183 | -0.01204 | 0.019997 | 0.006769 | 0.000709 | 0.000552 | -0.00379 | -0.0007 | -0.0013 |
| 390 | 0.031522 | -0.07215 | -0.01219 | 0.020142 | 0.005954 | 7.46E-05 | -0.00748 | 0.008648 | 0.001505 | -0.00036 |
| 391 | 0.031722 | -0.07108 | -0.01148 | 0.019173 | 0.004113 | -0.00103 | 0.003876 | 0.001522 | -0.00129 | 0.000525 |
| 392 | 0.031444 | -0.07115 | -0.01193 | 0.020658 | 0.00671 | 0.001003 | -0.00994 | 0.005916 | 0.001309 | 0.003316 |
| 393 | 0.030843 | -0.07164 | -0.01256 | 0.019816 | 0.00587 | -0.00075 | -0.00346 | 0.003521 | -0.001 | 0.001422 |
| 394 | 0.031385 | -0.07146 | -0.01178 | 0.020698 | 0.004265 | -0.0003 | -0.00148 | 0.000503 | -0.00094 | -0.00048 |
| 395 | 0.031164 | -0.07128 | -0.01172 | 0.01887 | 0.004262 | -0.00054 | 0.002166 | -0.00326 | 5.70E-05 | 0.003223 |
| 396 | 0.031515 | -0.07219 | -0.01155 | 0.020442 | 0.004829 | -0.00029 | -0.00128 | -0.00104 | 0.001379 | -0.00306 |
| 397 | 0.030708 | -0.07174 | -0.01227 | 0.01983 | 0.003404 | -0.00111 | 0.003374 | 0.001548 | -0.00299 | -0.00132 |
| 398 | 0.031674 | -0.07009 | -0.0107 | 0.017517 | 0.00296 | 0.003519 | -0.0001 | -0.00564 | 0.000746 | -0.00172 |
| 399 | 0.031404 | -0.07153 | -0.01172 | 0.018939 | 0.005839 | -0.00042 | 0.00608 | -0.00281 | 0.000105 | 0.001298 |
| 400 | 0.030528 | -0.0702 | -0.01167 | 0.018384 | 0.004423 | 0.00037 | 0.001639 | -0.00434 | 0.003108 | -0.00215 |
| 401 | 0.031043 | -0.07189 | -0.01259 | 0.020445 | 0.004065 | -0.00142 | 0.001489 | 0.000668 | -0.00275 | -0.00144 |
| 402 | 0.03072 | -0.07001 | -0.01194 | 0.017573 | 0.003497 | -0.00026 | -0.0019 | 0.001228 | -0.00023 | -0.00043 |
| 403 | 0.03133 | -0.07185 | -0.01203 | 0.02003 | 0.004592 | 0.000443 | -0.00611 | 0.001894 | 0.000149 | -0.00167 |
| 404 | 0.031088 | -0.07245 | -0.01242 | 0.019106 | 0.004732 | -0.00105 | 0.001311 | -0.00031 | 0.000991 | -0.00111 |
| 405 | 0.031021 | -0.07169 | -0.01225 | 0.020685 | 0.004108 | -0.00027 | 0.003386 | -0.00245 | 0.000124 | 1.08E-05 |
| 406 | 0.031196 | -0.07222 | -0.01214 | 0.020707 | 0.006281 | -0.00178 | 0.000429 | -0.00103 | -0.00198 | -0.00212 |
| 407 | 0.030927 | -0.07128 | -0.01179 | 0.017958 | 0.004741 | -0.00013 | -0.00068 | 0.000747 | -0.00102 | -0.0004 |
| 408 | 0.030854 | -0.07269 | -0.01256 | 0.020359 | 0.005856 | 0.000683 | -0.00142 | 0.002698 | -0.00065 | -0.00155 |
| 409 | 0.031827 | -0.0729 | -0.01259 | 0.022561 | 0.007662 | 0.000602 | -0.00179 | 0.000195 | -0.00157 | -0.00152 |
| 410 | 0.031247 | -0.07204 | -0.01231 | 0.020125 | 0.005042 | 0.001841 | -0.0076 | 0.00714 | -7.71E-05 | 5.82E-05 |
| 411 | 0.031052 | -0.07162 | -0.0121 | 0.020728 | 0.005986 | -0.00133 | 0.000985 | -0.00196 | -0.00286 | -0.00142 |
| 412 | 0.031608 | -0.07113 | -0.01195 | 0.019652 | 0.004476 | 0.001718 | -0.00686 | 0.005797 | -0.00075 | -0.00013 |
| 413 | 0.03148 | -0.07169 | -0.01172 | 0.019249 | 0.003508 | -0.00031 | 0.002671 | 0.000471 | -0.00128 | -0.00313 |
| 414 | 0.03125 | -0.07198 | -0.01194 | 0.019309 | 0.006655 | -0.00087 | 0.00197 | -0.0016 | -0.00087 | -0.00018 |
| 415 | 0.031674 | -0.07214 | -0.0125 | 0.020684 | 0.007081 | 0.000358 | 0.000809 | -0.00371 | 0.001068 | 0.000823 |
| 416 | 0.030974 | -0.07129 | -0.01213 | 0.017556 | 0.004818 | -0.00135 | -0.003 | 0.001093 | 0.000244 | -0.0022 |
| 417 | 0.031567 | -0.07226 | -0.0125 | 0.021731 | 0.005661 | 0.000328 | 0.002554 | -0.00285 | -0.00062 | 0.001924 |
| 418 | 0.031386 | -0.07101 | -0.01141 | 0.018636 | 0.004288 | 0.001108 | 0.002102 | -0.00113 | -0.00165 | -0.00099 |
| 419 | 0.031008 | -0.07051 | -0.01142 | 0.017086 | 0.003793 | -8.91E-06 | -0.00049 | 0.001044 | -0.00061 | -0.00041 |
| 420 | 0.030513 | -0.07018 | -0.01167 | 0.017531 | 0.003763 | -0.00013 | 0.000767 | -0.00174 | 0.000688 | -0.0001 |
| 421 | 0.031477 | -0.07197 | -0.01274 | 0.020226 | 0.005354 | -0.00103 | 0.001688 | -0.00353 | 0.000819 | 0.000571 |
| 422 | 0.031425 | -0.07211 | -0.01245 | 0.020104 | 0.005052 | -0.00085 | -0.00094 | 0.001591 | -0.00013 | 0.001773 |
| 423 | 0.031828 | -0.07278 | -0.01275 | 0.021296 | 0.006089 | 0.001483 | -0.00877 | 0.008109 | -0.00122 | 0.00206 |
| 424 | 0.031279 | -0.07154 | -0.01185 | 0.019763 | 0.0071 | 0.000494 | 0.002503 | 0.002003 | 0.000949 | 0.000517 |
| 425 | 0.031216 | -0.07132 | -0.01255 | 0.018773 | 0.004077 | -0.0011 | -0.00112 | 0.001854 | -0.00162 | -0.00159 |
| 426 | 0.030599 | -0.07183 | -0.01252 | 0.01965 | 0.005531 | -0.00079 | 0.002825 | -0.00321 | 0.002987 | -0.00118 |
| 427 | 0.031489 | -0.07237 | -0.0117 | 0.020732 | 0.005292 | 0.001679 | 0.002122 | -0.00208 | -0.00089 | -0.00124 |
| 428 | 0.031834 | -0.07176 | -0.0122 | 0.020519 | 0.006853 | 0.001184 | -0.00036 | 0.000103 | -0.00115 | 0.000479 |
| 429 | 0.030569 | -0.07159 | -0.01273 | 0.020074 | 0.0055 | -0.00057 | -0.00144 | 0.000136 | 0.000932 | 0.00255 |
| 430 | 0.03152 | -0.07287 | -0.01298 | 0.021653 | 0.006434 | 0.000189 | -0.01008 | 0.007132 | -0.00083 | 0.000329 |
| 431 | 0.031368 | -0.07169 | -0.01131 | 0.018502 | 0.005224 | -0.00144 | -0.00284 | -0.0017 | 0.000429 | 0.002734 |
| 432 | 0.03067 | -0.07178 | -0.0125 | 0.019581 | 0.004479 | 0.000336 | 0.002046 | -0.00064 | -0.00013 | -0.00116 |
| 433 | 0.030814 | -0.06937 | -0.01027 | 0.016322 | 0.002489 | 0.000919 | -0.00097 | 0.00024 | -0.00153 | 0.00087 |
| 434 | -0.04999 | -0.01903 | -0.01816 | -0.0913 | -0.01993 | -0.00095 | 0.055099 | -0.08732 | -0.01937 | 0.006166 |
| 435 | -0.05102 | -0.01742 | -0.01383 | -0.08797 | -0.01483 | 0.003019 | -0.0263 | 0.030484 | 0.011135 | 0.007465 |
| 436 | -0.05037 | -0.01616 | -0.01514 | -0.07603 | -0.01407 | 0.007154 | 0.043981 | -0.05644 | -0.02371 | -0.01855 |
| 437 | -0.05033 | -0.01578 | -0.01453 | -0.08551 | -0.01589 | -0.00504 | -0.0043 | 0.012172 | -0.00148 | 0.001395 |
| 438 | -0.05034 | -0.01644 | -0.01679 | -0.08425 | -0.02007 | -0.00493 | 0.058704 | -0.07025 | -0.01958 | -0.02027 |
| 439 | -0.05062 | -0.01719 | -0.01344 | -0.08693 | -0.01674 | -0.00327 | 0.023649 | -0.00389 | -0.01537 | 0.012904 |
| 440 | -0.05085 | -0.01768 | -0.01792 | -0.08946 | -0.03153 | 0.018924 | -0.22377 | 0.242144 | 0.022969 | -0.01485 |
| 441 | -0.04971 | -0.01299 | -0.01438 | -0.06506 | -0.01472 | 0.003978 | 0.019393 | -0.01558 | -0.00108 | -0.00138 |
| 442 | -0.05038 | -0.01863 | -0.01811 | -0.088 | -0.02417 | 0.00987 | 0.052569 | -0.04034 | -0.04656 | -0.02331 |
| 443 | -0.05019 | -0.01639 | -0.01653 | -0.08214 | -0.02396 | 0.00589 | 0.036486 | -0.03698 | 0.048226 | -0.01373 |
| 444 | -0.05089 | -0.01691 | -0.01512 | -0.08888 | -0.01714 | -0.0048 | -0.00464 | 0.008282 | 0.002462 | 0.020788 |
| 445 | -0.05031 | -0.01621 | -0.016 | -0.08793 | -0.0226 | -0.00462 | 0.019557 | -0.01492 | -0.00024 | 0.002414 |
| 446 | -0.05043 | -0.01506 | -0.01602 | -0.08234 | -0.02394 | -0.004 | -0.0182 | 0.016189 | -0.01418 | 0.017471 |
| 447 | -0.05041 | -0.01685 | -0.01652 | -0.08681 | -0.022 | -0.01014 | 0.028172 | -0.00611 | -0.01501 | 0.006074 |
| 448 | -0.05053 | -0.01585 | -0.01606 | -0.07924 | -0.02235 | -0.00998 | 0.049055 | -0.03875 | 0.035007 | -0.02774 |
| 449 | -0.0504 | -0.01729 | -0.01644 | -0.08837 | -0.02479 | 0.008621 | -0.05607 | 0.059781 | -0.00267 | -0.0051 |
| 450 | -0.0505 | -0.01548 | -0.01684 | -0.07707 | -0.01819 | -0.00074 | 0.037933 | -0.04496 | 0.017396 | 0.031347 |
| 451 | -0.05021 | -0.01768 | -0.01779 | -0.08511 | -0.02884 | 0.008624 | -0.21303 | 0.220382 | 0.029452 | -0.01983 |
| 452 | -0.05023 | -0.01705 | -0.01917 | -0.08825 | -0.02944 | -0.00072 | 0.009307 | -0.00285 | -0.01378 | 0.000465 |
| 453 | -0.04962 | -0.01778 | -0.01797 | -0.08298 | -0.02481 | -0.00326 | 0.037658 | -0.04816 | 0.048222 | -0.00618 |
| 454 | -0.04983 | -0.01808 | -0.01828 | -0.08851 | -0.02173 | -0.00071 | 0.026811 | -0.03968 | -0.00443 | -0.02355 |
| 455 | -0.05049 | -0.01727 | -0.01739 | -0.0868 | -0.02753 | -0.00631 | -0.02023 | 0.015952 | 0.064852 | -0.01736 |
| 456 | -0.05004 | -0.01749 | -0.01808 | -0.08279 | -0.02223 | 0.014576 | 0.049004 | -0.06264 | -0.01199 | -0.02918 |
| 457 | -0.0506 | -0.01832 | -0.01678 | -0.09168 | -0.02551 | -0.0058 | 0.052287 | -0.06292 | -0.00994 | 0.048411 |
| 458 | -0.05051 | -0.01809 | -0.0173 | -0.08972 | -0.02858 | 0.008786 | -0.22691 | 0.238235 | 0.028866 | -0.0161 |
| 459 | -0.05071 | -0.01919 | -0.01909 | -0.09246 | -0.03396 | 0.00996 | -0.22697 | 0.240722 | 0.042513 | -0.0213 |
| 460 | -0.05059 | -0.0178 | -0.01847 | -0.08854 | -0.02641 | 0.003493 | 0.054053 | -0.06736 | -0.02163 | 0.058893 |
| 461 | -0.05069 | -0.01739 | -0.01236 | -0.08337 | -0.01054 | 0.001521 | 0.039184 | -0.04226 | 0.006854 | 0.007803 |
| 462 | -0.04991 | -0.01768 | -0.01796 | -0.08697 | -0.0284 | 0.005616 | -0.19286 | 0.213984 | 0.036668 | -0.01931 |
| 463 | -0.05043 | -0.01459 | -0.01511 | -0.07344 | -0.01455 | -0.00437 | 0.057668 | -0.06655 | -0.0047 | 0.001006 |
| 464 | -0.05031 | -0.01442 | -0.01411 | -0.07079 | -0.01332 | 0.002003 | 0.042117 | -0.05793 | -0.02556 | -0.02088 |
| 465 | -0.05017 | -0.01396 | -0.01438 | -0.07744 | -0.0153 | 0.000446 | 0.035046 | -0.05672 | 0.000126 | 0.055635 |
| 466 | -0.04992 | -0.01538 | -0.01786 | -0.07662 | -0.02527 | -0.00127 | 0.019718 | -0.03458 | -0.02175 | -0.01087 |
| 467 | -0.05017 | -0.01641 | -0.01544 | -0.07829 | -0.01941 | 0.007358 | 0.002449 | 0.008673 | -0.01867 | -0.01948 |
| 468 | -0.05007 | -0.01964 | -0.01754 | -0.09352 | -0.02326 | 0.006993 | 0.065397 | -0.06498 | -0.01725 | 0.015623 |
| 469 | -0.04981 | -0.0155 | -0.01724 | -0.0797 | -0.0233 | -0.00042 | 0.029094 | -0.01569 | 0.009233 | -0.00654 |
| 470 | -0.05151 | -0.01802 | -0.01759 | -0.09268 | -0.03166 | 0.003047 | -0.19573 | 0.224623 | 0.023689 | -0.01539 |
| 471 | -0.05073 | -0.01518 | -0.01595 | -0.08403 | -0.02578 | -0.00947 | 0.050396 | -0.04863 | -0.00095 | 0.002528 |
| 472 | -0.05026 | -0.01825 | -0.01584 | -0.0841 | -0.01688 | 0.018055 | 0.000422 | -0.00039 | -0.0351 | -0.02533 |
| 473 | -0.05048 | -0.01579 | -0.01445 | -0.08526 | -0.01536 | -0.00759 | 0.032957 | -0.01709 | -0.02662 | 0.000164 |
| 474 | -0.05041 | -0.01852 | -0.0168 | -0.09655 | -0.02256 | -0.00865 | 0.03477 | -0.01486 | -0.0159 | 0.004774 |
| 475 | -0.04977 | -0.01756 | -0.01628 | -0.08306 | -0.0149 | 0.005659 | 0.047214 | -0.06905 | -0.03352 | 0.002927 |
| 476 | -0.05018 | -0.0119 | -0.01143 | -0.06687 | -0.00779 | -0.00095 | -0.01691 | 0.02954 | 0.004027 | 0.002144 |
| 477 | -0.05028 | -0.01715 | -0.01713 | -0.08726 | -0.01951 | -0.01022 | 0.073654 | -0.07985 | -0.02579 | 0.028824 |
| 478 | -0.04983 | -0.01722 | -0.01491 | -0.08459 | -0.01603 | 0.009465 | 0.007352 | -0.0194 | -0.0138 | -0.02522 |
| 479 | -0.0501 | -0.01854 | -0.01772 | -0.09044 | -0.02347 | 0.012853 | 0.034377 | -0.03155 | -0.03908 | -0.02697 |
| 480 | -0.05024 | -0.01691 | -0.01418 | -0.08053 | -0.01255 | 0.00932 | 0.049228 | -0.0593 | -0.01686 | -0.0109 |
| 481 | -0.05063 | -0.01898 | -0.0155 | -0.09346 | -0.02093 | -0.00055 | 0.056628 | -0.06034 | -0.03524 | 0.052597 |
| 482 | -0.05032 | -0.01743 | -0.01515 | -0.08837 | -0.02242 | 0.008733 | 0.037288 | -0.02818 | -0.0431 | -0.01094 |
| 483 | -0.05092 | -0.01514 | -0.01485 | -0.08267 | -0.02312 | -0.01177 | 0.026203 | 0.00095 | -0.00431 | 0.002333 |
| 484 | -0.05035 | -0.01481 | -0.01373 | -0.07485 | -0.01366 | 0.005844 | 0.068522 | -0.06555 | 0.0001 | 0.025132 |
| 485 | -0.05066 | -0.01757 | -0.01574 | -0.08691 | -0.02056 | 0.009709 | 0.05884 | -0.06285 | -0.0466 | -0.01929 |
| 486 | -0.05038 | -0.01775 | -0.01697 | -0.0918 | -0.02382 | 0.000528 | 0.043111 | -0.04681 | -0.01995 | 0.042671 |
| 487 | -0.04966 | -0.01132 | -0.01462 | -0.06428 | -0.02142 | -0.00081 | 0.008438 | -0.02125 | 0.012205 | -0.00648 |
| 488 | -0.04993 | -0.014 | -0.01534 | -0.07325 | -0.01736 | 0.002683 | 0.039837 | -0.03949 | 0.000511 | 0.005195 |
| 489 | -0.05063 | -0.01634 | -0.01572 | -0.081 | -0.01892 | -0.01189 | 0.029151 | -0.03353 | 0.056013 | -0.02316 |
| 490 | -0.05164 | -0.01841 | -0.01817 | -0.0941 | -0.03173 | -0.00797 | -0.16975 | 0.211099 | 0.024949 | 0.00391 |
| 491 | -0.05045 | -0.01701 | -0.01693 | -0.08739 | -0.02286 | -0.00568 | 0.016924 | 0.006358 | -0.00626 | 0.03439 |
| 492 | -0.05008 | -0.01409 | -0.01288 | -0.07404 | -0.01221 | -0.00104 | -0.04168 | 0.049508 | 0.007699 | 0.001735 |
| 493 | -0.0501 | -0.01685 | -0.0166 | -0.0841 | -0.02045 | -0.00645 | 0.079023 | -0.09133 | -0.01555 | 0.015916 |
| 494 | -0.04968 | -0.01623 | -0.01382 | -0.08213 | -0.01212 | 0.002619 | 0.016239 | -0.01106 | 0.004054 | -0.0057 |
| 495 | -0.05056 | -0.01677 | -0.01439 | -0.08171 | -0.01468 | 0.002078 | -0.0213 | 0.011328 | 0.021884 | -0.00572 |
| 496 | -0.05035 | -0.01445 | -0.01433 | -0.07718 | -0.0157 | -0.0103 | 0.022414 | -0.03272 | 0.000507 | -0.01388 |
| 497 | -0.04994 | -0.01839 | -0.01809 | -0.08863 | -0.02687 | -0.00071 | 0.045112 | -0.04352 | 0.060546 | -0.02486 |
| 498 | -0.04939 | -0.01418 | -0.01558 | -0.0694 | -0.02431 | -0.00355 | 0.001714 | -0.00701 | -0.01383 | -0.01693 |
| 499 | -0.05044 | -0.01808 | -0.01782 | -0.08654 | -0.02461 | -0.00276 | 0.052458 | -0.06462 | -0.01195 | 0.032331 |
| 500 | -0.04969 | -0.01558 | -0.01612 | -0.07899 | -0.02157 | 0.005202 | 0.017785 | -0.03765 | 0.01149 | 0.003955 |
| 501 | -0.04987 | -0.01605 | -0.01598 | -0.08081 | -0.01994 | -0.00048 | 0.041421 | -0.07276 | -0.01252 | 0.026539 |
| 502 | -0.0509 | -0.01698 | -0.01663 | -0.08548 | -0.02215 | -0.00096 | 0.011998 | -0.00614 | -0.00013 | -0.00483 |
| 503 | -0.05055 | -0.01611 | -0.01747 | -0.08408 | -0.02459 | -0.00346 | 0.024288 | -0.01837 | -0.00981 | -0.00258 |
| 504 | -0.05044 | -0.01797 | -0.01823 | -0.09277 | -0.03068 | -0.00508 | 0.008416 | -0.00436 | -0.0227 | 0.003348 |
| 505 | -0.05034 | -0.01715 | -0.01786 | -0.08355 | -0.02122 | 0.003911 | 0.029862 | -0.01857 | -0.00774 | -0.01245 |
| 506 | -0.05109 | -0.01638 | -0.0138 | -0.08453 | -0.01407 | 0.00392 | -0.02347 | 0.029842 | -7.40E-05 | -0.01436 |
| 507 | -0.05006 | -0.0165 | -0.01609 | -0.07903 | -0.02321 | -0.00799 | -0.00426 | 0.025151 | 0.018807 | -0.00429 |
| 508 | -0.05059 | -0.01592 | -0.01576 | -0.08107 | -0.02395 | 0.01001 | -0.18003 | 0.194336 | 0.032752 | -0.0124 |
| 509 | -0.05062 | -0.01719 | -0.01786 | -0.09058 | -0.02839 | -0.00149 | -0.06679 | 0.059989 | 0.029613 | -0.00387 |
| 510 | -0.04995 | -0.01647 | -0.01709 | -0.08075 | -0.02407 | 0.012843 | 0.035783 | -0.02604 | -0.01492 | -0.00639 |
| 511 | -0.04969 | -0.01577 | -0.01659 | -0.08194 | -0.02034 | 0.008465 | 0.010902 | -0.01249 | -0.01198 | 0.00196 |
| 512 | -0.04976 | -0.01385 | -0.01545 | -0.06659 | -0.01935 | -0.0088 | -0.00392 | 0.010083 | 0.028637 | -0.00986 |
| 513 | -0.05126 | -0.01634 | -0.01276 | -0.08317 | -0.00959 | -0.00855 | 0.042821 | -0.01388 | 0.00389 | 0.003033 |
