# Supplementary material for: A high-density genome-wide association with absolute blood monocyte count in domestic sheep identifies novel loci
Source: PLoS One. 2022 May 6;17(5):e0266748. doi: 10.1371/journal.pone.0266748 (PMC9075649; doi:10.1371/journal.pone.0266748)
Supplement: S2 Table — The absolute monocyte count matched to sheep study ID. (DOCX) [file pone.0266748.s008.docx]

**Table S2. Monocyte counts by study ID.**

| **Sheep** | **Breed** | **Absolute monocyte count** |
| --- | --- | --- |
| 1 | Columbia | 350 |
| 2 | Columbia | 200 |
| 3 | Columbia | 90 |
| 4 | Columbia | 70 |
| 5 | Columbia | 120 |
| 6 | Columbia | 210 |
| 7 | Columbia | 90 |
| 8 | Columbia | 180 |
| 9 | Columbia | 410 |
| 10 | Columbia | 70 |
| 11 | Columbia | 120 |
| 12 | Columbia | 220 |
| 13 | Columbia | 250 |
| 14 | Columbia | 190 |
| 15 | Columbia | 120 |
| 16 | Columbia | 100 |
| 17 | Columbia | 230 |
| 18 | Columbia | 100 |
| 19 | Columbia | 380 |
| 20 | Columbia | 120 |
| 21 | Columbia | 570 |
| 22 | Columbia | 100 |
| 23 | Columbia | 180 |
| 24 | Columbia | 210 |
| 25 | Columbia | 320 |
| 26 | Columbia | 190 |
| 27 | Columbia | 220 |
| 28 | Columbia | 270 |
| 29 | Columbia | 40 |
| 30 | Columbia | 50 |
| 31 | Columbia | 420 |
| 32 | Columbia | 320 |
| 33 | Columbia | 460 |
| 34 | Columbia | 290 |
| 35 | Columbia | 80 |
| 36 | Columbia | 120 |
| 37 | Columbia | 360 |
| 38 | Columbia | 80 |
| 39 | Columbia | 660 |
| 40 | Columbia | 120 |
| 41 | Columbia | 160 |
| 42 | Columbia | 100 |
| 43 | Columbia | 150 |
| 44 | Columbia | 160 |
| 45 | Columbia | 90 |
| 46 | Columbia | 0 |
| 47 | Columbia | 350 |
| 48 | Columbia | 50 |
| 49 | Columbia | 350 |
| 50 | Columbia | 880 |
| 51 | Columbia | 240 |
| 52 | Columbia | 110 |
| 53 | Columbia | 220 |
| 54 | Columbia | 390 |
| 55 | Columbia | 230 |
| 56 | Columbia | 220 |
| 57 | Columbia | 240 |
| 58 | Columbia | 180 |
| 59 | Columbia | 260 |
| 60 | Columbia | 280 |
| 61 | Columbia | 260 |
| 62 | Columbia | 550 |
| 63 | Columbia | 520 |
| 64 | Columbia | 70 |
| 65 | Columbia | 290 |
| 66 | Columbia | 120 |
| 67 | Columbia | 260 |
| 68 | Rambouillet | 192 |
| 69 | Rambouillet | 220 |
| 70 | Rambouillet | 340 |
| 71 | Rambouillet | 180 |
| 72 | Rambouillet | 220 |
| 73 | Rambouillet | 200 |
| 74 | Rambouillet | 80 |
| 75 | Rambouillet | 50 |
| 76 | Rambouillet | 80 |
| 77 | Rambouillet | 50 |
| 78 | Rambouillet | 120 |
| 79 | Rambouillet | 320 |
| 80 | Rambouillet | 140 |
| 81 | Rambouillet | 480 |
| 82 | Rambouillet | 140 |
| 83 | Rambouillet | 230 |
| 84 | Rambouillet | 430 |
| 85 | Rambouillet | 240 |
| 86 | Rambouillet | 230 |
| 87 | Rambouillet | 360 |
| 88 | Rambouillet | 320 |
| 89 | Rambouillet | 310 |
| 90 | Rambouillet | 280 |
| 91 | Rambouillet | 680 |
| 92 | Rambouillet | 50 |
| 93 | Rambouillet | 280 |
| 94 | Rambouillet | 40 |
| 95 | Rambouillet | 80 |
| 96 | Rambouillet | 310 |
| 97 | Rambouillet | 100 |
| 98 | Rambouillet | 200 |
| 99 | Rambouillet | 230 |
| 100 | Rambouillet | 260 |
| 101 | Rambouillet | 130 |
| 102 | Rambouillet | 60 |
| 103 | Rambouillet | 160 |
| 104 | Rambouillet | 140 |
| 105 | Rambouillet | 140 |
| 106 | Rambouillet | 210 |
| 107 | Rambouillet | 190 |
| 108 | Rambouillet | 200 |
| 109 | Rambouillet | 230 |
| 110 | Rambouillet | 230 |
| 111 | Rambouillet | 340 |
| 112 | Rambouillet | 170 |
| 113 | Rambouillet | 260 |
| 114 | Rambouillet | 100 |
| 115 | Rambouillet | 200 |
| 116 | Rambouillet | 270 |
| 117 | Rambouillet | 260 |
| 118 | Rambouillet | 80 |
| 119 | Rambouillet | 570 |
| 120 | Rambouillet | 110 |
| 121 | Rambouillet | 130 |
| 122 | Rambouillet | 50 |
| 123 | Rambouillet | 90 |
| 124 | Rambouillet | 40 |
| 125 | Rambouillet | 120 |
| 126 | Rambouillet | 290 |
| 127 | Rambouillet | 170 |
| 128 | Rambouillet | 150 |
| 129 | Rambouillet | 180 |
| 130 | Rambouillet | 170 |
| 131 | Rambouillet | 60 |
| 132 | Rambouillet | 50 |
| 133 | Rambouillet | 80 |
| 134 | Rambouillet | 250 |
| 135 | Rambouillet | 180 |
| 136 | Rambouillet | 130 |
| 137 | Rambouillet | 290 |
| 138 | Rambouillet | 130 |
| 139 | Rambouillet | 310 |
| 140 | Rambouillet | 300 |
| 141 | Rambouillet | 120 |
| 142 | Rambouillet | 140 |
| 143 | Rambouillet | 140 |
| 144 | Rambouillet | 170 |
| 145 | Rambouillet | 80 |
| 146 | Rambouillet | 100 |
| 147 | Rambouillet | 140 |
| 148 | Rambouillet | 190 |
| 149 | Rambouillet | 140 |
| 150 | Rambouillet | 50 |
| 151 | Rambouillet | 170 |
| 152 | Rambouillet | 220 |
| 153 | Rambouillet | 150 |
| 154 | Rambouillet | 270 |
| 155 | Rambouillet | 0 |
| 156 | Rambouillet | 260 |
| 157 | Rambouillet | 170 |
| 158 | Rambouillet | 130 |
| 159 | Rambouillet | 30 |
| 160 | Rambouillet | 150 |
| 161 | Rambouillet | 270 |
| 162 | Rambouillet | 140 |
| 163 | Rambouillet | 70 |
| 164 | Rambouillet | 150 |
| 165 | Rambouillet | 80 |
| 166 | Rambouillet | 130 |
| 167 | Rambouillet | 110 |
| 168 | Rambouillet | 100 |
| 169 | Rambouillet | 210 |
| 170 | Rambouillet | 120 |
| 171 | Rambouillet | 210 |
| 172 | Rambouillet | 230 |
| 173 | Rambouillet | 60 |
| 174 | Rambouillet | 410 |
| 175 | Rambouillet | 260 |
| 176 | Rambouillet | 170 |
| 177 | Rambouillet | 160 |
| 178 | Rambouillet | 270 |
| 179 | Rambouillet | 130 |
| 180 | Rambouillet | 110 |
| 181 | Rambouillet | 0 |
| 182 | Rambouillet | 170 |
| 183 | Rambouillet | 150 |
| 184 | Rambouillet | 120 |
| 185 | Rambouillet | 120 |
| 186 | Rambouillet | 150 |
| 187 | Rambouillet | 110 |
| 188 | Rambouillet | 190 |
| 189 | Rambouillet | 500 |
| 190 | Rambouillet | 70 |
| 191 | Rambouillet | 120 |
| 192 | Rambouillet | 220 |
| 193 | Rambouillet | 200 |
| 194 | Rambouillet | 250 |
| 195 | Rambouillet | 110 |
| 196 | Rambouillet | 130 |
| 197 | Rambouillet | 260 |
| 198 | Rambouillet | 100 |
| 199 | Rambouillet | 170 |
| 200 | Rambouillet | 140 |
| 201 | Rambouillet | 340 |
| 202 | Rambouillet | 130 |
| 203 | Rambouillet | 300 |
| 204 | Rambouillet | 170 |
| 205 | Rambouillet | 530 |
| 206 | Rambouillet | 230 |
| 207 | Rambouillet | 240 |
| 208 | Rambouillet | 340 |
| 209 | Rambouillet | 110 |
| 210 | Rambouillet | 100 |
| 211 | Rambouillet | 200 |
| 212 | Rambouillet | 0 |
| 213 | Rambouillet | 380 |
| 214 | Rambouillet | 90 |
| 215 | Rambouillet | 120 |
| 216 | Rambouillet | 60 |
| 217 | Rambouillet | 140 |
| 218 | Rambouillet | 290 |
| 219 | Rambouillet | 90 |
| 220 | Rambouillet | 190 |
| 221 | Rambouillet | 240 |
| 222 | Rambouillet | 200 |
| 223 | Rambouillet | 290 |
| 224 | Rambouillet | 120 |
| 225 | Rambouillet | 90 |
| 226 | Rambouillet | 90 |
| 227 | Rambouillet | 250 |
| 228 | Rambouillet | 150 |
| 229 | Rambouillet | 200 |
| 230 | Rambouillet | 400 |
| 231 | Rambouillet | 150 |
| 232 | Rambouillet | 150 |
| 233 | Rambouillet | 60 |
| 234 | Rambouillet | 330 |
| 235 | Rambouillet | 200 |
| 236 | Rambouillet | 160 |
| 237 | Rambouillet | 60 |
| 238 | Rambouillet | 20 |
| 239 | Rambouillet | 140 |
| 240 | Rambouillet | 140 |
| 241 | Rambouillet | 190 |
| 242 | Rambouillet | 60 |
| 243 | Rambouillet | 90 |
| 244 | Rambouillet | 50 |
| 245 | Rambouillet | 60 |
| 246 | Rambouillet | 240 |
| 247 | Rambouillet | 240 |
| 248 | Rambouillet | 270 |
| 249 | Rambouillet | 290 |
| 250 | Rambouillet | 40 |
| 251 | Rambouillet | 180 |
| 252 | Rambouillet | 590 |
| 253 | Rambouillet | 250 |
| 254 | Rambouillet | 280 |
| 255 | Rambouillet | 280 |
| 256 | Rambouillet | 170 |
| 257 | Rambouillet | 330 |
| 258 | Rambouillet | 450 |
| 259 | Rambouillet | 170 |
| 260 | Rambouillet | 450 |
| 261 | Rambouillet | 100 |
| 262 | Rambouillet | 310 |
| 263 | Rambouillet | 570 |
| 264 | Rambouillet | 100 |
| 265 | Rambouillet | 220 |
| 266 | Rambouillet | 170 |
| 267 | Rambouillet | 700 |
| 268 | Rambouillet | 90 |
| 269 | Rambouillet | 320 |
| 270 | Rambouillet | 220 |
| 271 | Rambouillet | 210 |
| 272 | Rambouillet | 70 |
| 273 | Rambouillet | 90 |
| 274 | Rambouillet | 190 |
| 275 | Rambouillet | 40 |
| 276 | Rambouillet | 180 |
| 277 | Rambouillet | 150 |
| 278 | Rambouillet | 260 |
| 279 | Rambouillet | 120 |
| 280 | Rambouillet | 80 |
| 281 | Rambouillet | 220 |
| 282 | Rambouillet | 70 |
| 283 | Rambouillet | 170 |
| 284 | Rambouillet | 430 |
| 285 | Rambouillet | 350 |
| 286 | Rambouillet | 370 |
| 287 | Rambouillet | 360 |
| 288 | Rambouillet | 440 |
| 289 | Rambouillet | 150 |
| 290 | Rambouillet | 0 |
| 291 | Rambouillet | 270 |
| 292 | Rambouillet | 140 |
| 293 | Rambouillet | 170 |
| 294 | Rambouillet | 100 |
| 295 | Rambouillet | 190 |
| 296 | Rambouillet | 400 |
| 297 | Rambouillet | 50 |
| 298 | Rambouillet | 270 |
| 299 | Rambouillet | 40 |
| 300 | Rambouillet | 160 |
| 301 | Rambouillet | 150 |
| 302 | Rambouillet | 90 |
| 303 | Rambouillet | 280 |
| 304 | Rambouillet | 0 |
| 305 | Rambouillet | 240 |
| 306 | Rambouillet | 270 |
| 307 | Rambouillet | 180 |
| 308 | Rambouillet | 220 |
| 309 | Rambouillet | 420 |
| 310 | Rambouillet | 220 |
| 311 | Rambouillet | 50 |
| 312 | Rambouillet | 0 |
| 313 | Rambouillet | 200 |
| 314 | Rambouillet | 40 |
| 315 | Rambouillet | 340 |
| 316 | Rambouillet | 190 |
| 317 | Rambouillet | 330 |
| 318 | Polypay | 200 |
| 319 | Polypay | 320 |
| 320 | Polypay | 210 |
| 321 | Polypay | 80 |
| 322 | Polypay | 340 |
| 323 | Polypay | 180 |
| 324 | Polypay | 60 |
| 325 | Polypay | 260 |
| 326 | Polypay | 240 |
| 327 | Polypay | 260 |
| 328 | Polypay | 530 |
| 329 | Polypay | 310 |
| 330 | Polypay | 200 |
| 331 | Polypay | 130 |
| 332 | Polypay | 230 |
| 333 | Polypay | 90 |
| 334 | Polypay | 120 |
| 335 | Polypay | 160 |
| 336 | Polypay | 220 |
| 337 | Polypay | 410 |
| 338 | Polypay | 330 |
| 339 | Polypay | 110 |
| 340 | Polypay | 120 |
| 341 | Polypay | 130 |
| 342 | Polypay | 120 |
| 343 | Polypay | 60 |
| 344 | Polypay | 80 |
| 345 | Polypay | 320 |
| 346 | Polypay | 250 |
| 347 | Polypay | 400 |
| 348 | Polypay | 60 |
| 349 | Polypay | 210 |
| 350 | Polypay | 40 |
| 351 | Polypay | 140 |
| 352 | Polypay | 390 |
| 353 | Polypay | 260 |
| 354 | Polypay | 230 |
| 355 | Polypay | 210 |
| 356 | Polypay | 140 |
| 357 | Polypay | 340 |
| 358 | Polypay | 90 |
| 359 | Polypay | 160 |
| 360 | Polypay | 170 |
| 361 | Polypay | 270 |
| 362 | Polypay | 350 |
| 363 | Polypay | 140 |
| 364 | Polypay | 310 |
| 365 | Polypay | 270 |
| 366 | Polypay | 200 |
| 367 | Polypay | 60 |
| 368 | Polypay | 140 |
| 369 | Polypay | 130 |
| 370 | Polypay | 360 |
| 371 | Polypay | 160 |
| 372 | Polypay | 200 |
| 373 | Polypay | 180 |
| 374 | Polypay | 370 |
| 375 | Polypay | 140 |
| 376 | Polypay | 200 |
| 377 | Polypay | 340 |
| 378 | Polypay | 0 |
| 379 | Polypay | 310 |
| 380 | Polypay | 340 |
| 381 | Polypay | 260 |
| 382 | Polypay | 110 |
| 383 | Polypay | 200 |
| 384 | Polypay | 130 |
| 385 | Polypay | 160 |
| 386 | Polypay | 240 |
| 387 | Polypay | 260 |
| 388 | Polypay | 140 |
| 389 | Polypay | 260 |
| 390 | Polypay | 120 |
| 391 | Polypay | 150 |
| 392 | Polypay | 330 |
| 393 | Polypay | 170 |
| 394 | Polypay | 200 |
| 395 | Polypay | 80 |
| 396 | Polypay | 140 |
| 397 | Polypay | 100 |
| 398 | Polypay | 370 |
| 399 | Polypay | 400 |
| 400 | Polypay | 210 |
| 401 | Polypay | 280 |
| 402 | Polypay | 90 |
| 403 | Polypay | 140 |
| 404 | Polypay | 220 |
| 405 | Polypay | 200 |
| 406 | Polypay | 50 |
| 407 | Polypay | 120 |
| 408 | Polypay | 90 |
| 409 | Polypay | 200 |
| 410 | Polypay | 360 |
| 411 | Polypay | 190 |
| 412 | Polypay | 40 |
| 413 | Polypay | 210 |
| 414 | Polypay | 60 |
| 415 | Polypay | 220 |
| 416 | Polypay | 430 |
| 417 | Polypay | 290 |
| 418 | Polypay | 280 |
| 419 | Polypay | 230 |
| 420 | Polypay | 190 |
| 421 | Polypay | 80 |
| 422 | Polypay | 510 |
| 423 | Polypay | 150 |
| 424 | Polypay | 240 |
| 425 | Polypay | 150 |
| 426 | Polypay | 220 |
| 427 | Polypay | 80 |
| 428 | Polypay | 70 |
| 429 | Polypay | 310 |
| 430 | Polypay | 150 |
| 431 | Polypay | 270 |
| 432 | Polypay | 50 |
| 433 | Polypay | 110 |
| 434 | Polypay | 150 |
| 435 | Polypay | 140 |
| 436 | Polypay | 100 |
| 437 | Polypay | 290 |
| 438 | Polypay | 360 |
| 439 | Polypay | 150 |
| 440 | Polypay | 380 |
| 441 | Polypay | 220 |
| 442 | Polypay | 0 |
| 443 | Polypay | 160 |
| 444 | Polypay | 160 |
| 445 | Polypay | 350 |
| 446 | Polypay | 120 |
| 447 | Polypay | 50 |
| 448 | Polypay | 90 |
| 449 | Polypay | 180 |
| 450 | Polypay | 250 |
| 451 | Polypay | 180 |
| 452 | Polypay | 290 |
| 453 | Polypay | 270 |
| 454 | Polypay | 280 |
| 455 | Polypay | 510 |
| 456 | Polypay | 370 |
| 457 | Polypay | 460 |
| 458 | Polypay | 420 |
| 459 | Polypay | 420 |
| 460 | Polypay | 90 |
| 461 | Polypay | 40 |
| 462 | Polypay | 70 |
| 463 | Polypay | 230 |
| 464 | Polypay | 140 |
| 465 | Polypay | 140 |
| 466 | Polypay | 300 |
| 467 | Polypay | 250 |
| 468 | Polypay | 450 |
| 469 | Polypay | 110 |
| 470 | Polypay | 180 |
| 471 | Polypay | 340 |
| 472 | Polypay | 250 |
| 473 | Polypay | 230 |
| 474 | Polypay | 230 |
| 475 | Polypay | 390 |
| 476 | Polypay | 150 |
| 477 | Polypay | 330 |
| 478 | Polypay | 150 |
| 479 | Polypay | 230 |
| 480 | Polypay | 60 |
| 481 | Polypay | 230 |
| 482 | Polypay | 260 |
| 483 | Polypay | 360 |
| 484 | Polypay | 180 |
| 485 | Polypay | 150 |
| 486 | Polypay | 260 |
| 487 | Polypay | 390 |
| 488 | Polypay | 340 |
| 489 | Polypay | 350 |
| 490 | Polypay | 150 |
| 491 | Polypay | 330 |
| 492 | Polypay | 210 |
| 493 | Polypay | 50 |
| 494 | Polypay | 140 |
| 495 | Polypay | 100 |
| 496 | Polypay | 90 |
| 497 | Polypay | 240 |
| 498 | Polypay | 260 |
| 499 | Polypay | 360 |
| 500 | Polypay | 80 |
| 501 | Polypay | 210 |
| 502 | Polypay | 260 |
| 503 | Polypay | 140 |
| 504 | Polypay | 460 |
| 505 | Polypay | 100 |
| 506 | Polypay | 140 |
| 507 | Polypay | 510 |
| 508 | Polypay | 130 |
| 509 | Polypay | 170 |
| 510 | Polypay | 270 |
| 511 | Polypay | 300 |
| 512 | Polypay | 420 |
| 513 | Polypay | 0 |
