# Supplementary material for: A high-density genome-wide association with absolute blood monocyte count in domestic sheep identifies novel loci
Source: PLoS One. 2022 May 6;17(5):e0266748. doi: 10.1371/journal.pone.0266748 (PMC9075649; doi:10.1371/journal.pone.0266748)
Supplement: S3 Table — The distribution characteristics for the absolute monocyte counts are summarized. (DOCX) [file pone.0266748.s009.docx]

**Table S3. Distribution characteristics of monocyte counts.**

| **Measure** | **Value** |
| --- | --- |
| Minimum | 0 |
| Maximum | 880 |
| Mean | 205.7 |
| Median | 190 |
| Mode | 140 |
| Standard deviation | 125.0 |
| Skewness | 1.148 |
| Kurtosis | 2.332 |
