# Supplementary material for: A high-density genome-wide association with absolute blood monocyte count in domestic sheep identifies novel loci
Source: PLoS One. 2022 May 6;17(5):e0266748. doi: 10.1371/journal.pone.0266748 (PMC9075649; doi:10.1371/journal.pone.0266748)
Supplement: S4 Table — +Allele associated with higher absolute monocyte count. (DOCX) [file pone.0266748.s010.docx]

**Table S4. Allele frequencies of the top SNPs by breed.**

| **refSNP** | **A1** | **A2** | **MAF (Columbia)** | **MAF (Rambouillet)** | **MAF (Polypay)** |
| --- | --- | --- | --- | --- | --- |
| *rs401041089* | G | A^+^ | 0.082 | 0 | 0 |
| *rs428401450* | G | T^+^ | 0.201 | 0.152 | 0.132 |
| *rs425174370* | T | G^+^ | 0.067 | 0 | 0.013 |
| *rs399452398* | C | T^+^ | 0.082 | 0.054 | 0.002 |
| *rs429734375* | A | G^+^ | 0.261 | 0.162 | 0.222 |
| *rs421879522* | C | T^+^ | 0.007 | 0.082 | 0.064 |
| *rs428909416* | G^+^ | A | 0.470 | 0.492 | 0.278 |
| *rs414400434* | T^+^ | C | 0.440 | 0.492 | 0.548 |
| *rs399619443* | C^+^ | T | 0.082 | 0.072 | 0.077 |
| *rs418310516* | C | A^+^ | 0.127 | 0.032 | 0.110 |
| *rs427185509* | T | C^+^ | 0.127 | 0.004 | 0.018 |
| *rs423783355* | A | G^+^ | 0.142 | 0.028 | 0.003 |

^+^Allele associated with higher absolute monocyte count.
